# Some Muirhead Mean Operators for Intuitionistic Fuzzy Numbers and Their Applications to Group Decision Making

**DOI:** 10.1371/journal.pone.0168767

**Published:** 2017-01-19

**Authors:** Peide Liu, Dengfeng Li

**Affiliations:** 1 School of Economics and Management, Fuzhou University, Fuzhou, Fujian, China; 2 School of Management Science and Engineering, Shandong University of Finance and Economics, Jinan, Shandong, China; Jiangnan University, CHINA

## Abstract

Muirhead mean (MM) is a well-known aggregation operator which can consider interrelationships among any number of arguments assigned by a variable vector. Besides, it is a universal operator since it can contain other general operators by assigning some special parameter values. However, the MM can only process the crisp numbers. Inspired by the MM’ advantages, the aim of this paper is to extend MM to process the intuitionistic fuzzy numbers (IFNs) and then to solve the multi-attribute group decision making (MAGDM) problems. Firstly, we develop some intuitionistic fuzzy Muirhead mean (IFMM) operators by extending MM to intuitionistic fuzzy information. Then, we prove some properties and discuss some special cases with respect to the parameter vector. Moreover, we present two new methods to deal with MAGDM problems with the intuitionistic fuzzy information based on the proposed MM operators. Finally, we verify the validity and reliability of our methods by using an application example, and analyze the advantages of our methods by comparing with other existing methods.

## Introduction

Multi-attribute decision making (MADM) and MAGDM are the important aspects of decision sciences, and they can give the ranking results for the finite alternatives or select best choice from them according to the attribute values of different alternatives [1]. In the decision process, an important problem is how to express the attribute value. Because of the complexity of decision making problems, sometimes it is difficult to describe the attributes by crisp numbers. The intuitionistic fuzzy set (IFS), proposed by Atanassov [2,3], is a very effective tool to express the complex fuzzy information because it has the significant advantages with the membership and non-membership functions, so since it was proposed, a lot of research achievements about theory and methods have been made, and it has three aspects: (1) the basic theory, such as the operational laws [2–4], comparison method [5–7], distance [8], similarity degree [9,10], correlation measure [11], information entropy [12], and so on; (2) the extended traditional MADM and MAGDM methods for IFS, such as TOPSIS method [13,14], VIKOR method [15], GRA method [16], PROMETHE [17], ELECTRE [18], TODIM method [19], DEMATEL method [20] and so on; (3) the MADM and MAGDM methods based on intuitionistic fuzzy aggregation operators.

The aggregation operators can aggregate the evaluation information of individual decision maker to collective one, and/or aggregate all attribute values to the comprehensive value, then to rank the alternatives by the comprehensive value of alternatives, while the extended traditional decision methods can only rank the alternatives based on some decision principles, for example, TOPSIS can give the ranking results based on the relative closeness. Obviously, the methods based on aggregation operators are better than the extended traditional decision methods because they can provide two aspects of information, including the comprehensive values of the alternatives and the ranking results while the extended traditional decision methods can only give ranking information. So a large number of aggregation operators are extended to process the different fuzzy information [21–26], and then they are applied to solve the MADM or MDGDM problems. Especially, because IFS can easily express the fuzzy information, the researches on intuitionistic fuzzy aggregation operators have made many achievements, for example, arithmetic and geometric operators for IFNs [27,28], distance operator [29], neutral averaging operators [30], generalized operators [31], and so on. Except this basic function of aggregation operators which can aggregate a collection of data to one, some aggregation operators can achieve some special functions. For example, power operators [25,26,32,33] and dependent aggregation operators [34,35] which can relieve the influences of some unreasonable data give by biased decision makers, Bonferroni mean (BM) [36–39] and the Heronian mean (HM) [24,40,41], which consider interrelationships of aggregated arguments.

In real decision making, there exist the interrelationships among the attributes in MADM or MAGDM problems. BM or HM operators provided a tool to consider the interrelationships of aggregated arguments; however, they can only consider the interrelationships between two attributes and cannot process the interrelationships among three or more than three attributes. Muirhead mean (MM) [42] is a well-known aggregation operator which can consider interrelationships among any number of arguments assigned by a variable vector, and some existing operators, such as arithmetic and geometric operators (not considering the interrelationships), BM operator and Maclaurin symmetric mean [43], are the special cases of MM operator. Therefore, the MM can offer a flexible and robust mechanism to process the information fusion problem and make it more adequate to solve MADM or MAGDM problems. However, the original MM can only deal with the numeric arguments, in order to make the MM operator to process the fuzzy information, Qin and Liu [44] extended the MM operator to process the 2-tuple linguistic information, and proposed some 2-tuple linguistic MM operators, then applied them to solve the MAGDM problems.

Because IFNs can easily describe the fuzzy information, and the MM can capture interrelationships among any number of arguments assigned by a variable vector, it is necessary to extend the MM operator to deal with the IFNs. The goal of this paper is to propose some intuitionistic fuzzy MM operators by extending MM to intuitionistic fuzzy information, then to study some properties and some special cases of these operators, and applied them to solve the MADM or MAGDM problems in which the attributes take the form of IFNs.

In order to achieve this goal, the rest of this paper is organized as follows. Section 2 reviews some basic concepts and theory. In Section 3, we propose the some intuitionistic fuzzy MM operators, and study some properties and some special cases of these operators. In Section 4, we develop two MAGDM methods for IFNs based on the proposed intuitionistic fuzzy MM operators. In Section 5, an illustrative example is given to verify the validity of the proposed methods and to show their advantages. In Section 6, we give some conclusions of this study.

## Preliminaries

In this part, we briefly introduce some basic concepts about IFNs and MM operator so that the readers can easily understand this study.

### A. IFSs

**Definition 1 [2,3].** Let *Y* = {*y*_1_,*y*_2_,⋯,*y*_*n*_} be a universe of discourse. An IFS *X* in *Y* is defined by

$$X=\{<y,u_{X}(y),v_{X}(y)>|y\in Y\},$$ (1)

where, *u*_*X*_(*y*),*v*_*X*_(*y*) ∈ [0,1] and meets the condition 0 ≤ *u*_*X*_(*y*)+*v*_*X*_(*y*) ≤ 1, ∀*y* ∈ *Y*. *u*_*X*_(*y*) and *v*_*X*_(*y*) are the membership function and non-membership function of the element *y* to the set *X*, respectively, and *π*(*y*) = 1− *u*_*X*_(*y*)−*v*_*X*_(*y*), ∀*y* ∈ *Y* is called indeterminacy degree of *y* to the set *X*. Obviously, it meets 0 ≤ *π*(*y*) ≤ 1, ∀*y* ∈ *Y*.

Further, Xu and Xia [45] called (*u*_*X*_(*y*),*v*_*X*_(*y*)) an intuitionistic fuzzy number (IFN). For convenience, it was expressed as $\tilde{x}=\left(u_{\tilde{x}},v_{\tilde{x}}\right)$ with the conditions $u_{\tilde{x}}\in [0,1]$, $v_{\tilde{x}}\in [0,1]$ and $0\leq u_{\tilde{x}}+v_{\tilde{x}}\leq 1$.

Let ${\tilde{x}}_{1}=\left(x_{1},y_{1}\right)$ and ${\tilde{x}}_{2}=\left(x_{2},y_{2}\right)$ be two IFNs, and *λ* > 0, then the operational rules were defined as follows [3]:

$$(i){\tilde{x}}_{1}⊕{\tilde{x}}_{2}=\left(x_{1}+x_{2}-x_{1}x_{2},y_{1}y_{2}\right),$$ (2)

$$(ii)\;{\tilde{x}}_{1}⊗{\tilde{x}}_{2}=\left(x_{1}x_{2},y_{1}+y_{2}-y_{1}y_{2}\right),$$ (3)

$$(iii)\;\lambda {\tilde{x}}_{1}=\left(1-{\left(1-x_{1}\right)}^{\lambda },y_{1}{}^{\lambda }\right),$$ (4)

$$(iv)\;{\tilde{x}}_{1}^{\lambda }=\left(x_{1}{}^{\lambda },1-{\left(1-y_{1}\right)}^{\lambda }\right).$$ (5)

In the following, we give some definitions so as to compare the IFNs.

**Definition 2 [5].** Let $\tilde{x}=\left(u_{\tilde{x}},v_{\tilde{x}}\right)$ be an IFN, then a score function *S* of $\tilde{x}$ is shown as follows:

$$S(\tilde{x})=u_{\tilde{x}}-v_{\tilde{x}},$$ (6)

Obviously, $S(\tilde{x})\in \left[-1,1\right]$. The larger the score $S(\tilde{x})$ is,the greater the IFN $\tilde{x}$ is.

**Definition 3 [6].** Let $\tilde{x}=\left(u_{\tilde{x}},v_{\tilde{x}}\right)$ be an IFN, then the accuracy function *H* of $\tilde{x}$ is defined as follows:

$$H(\tilde{x})=u_{\tilde{x}}+v_{\tilde{x}},$$ (7)

where $H(\tilde{x})\in \left[0,1\right]$. The larger the accuracy degree $H(\tilde{x})$ is, the greater $\tilde{x}$ is.

Based on the above score function and the accuracy function, a comparison method of IFNs was proposed by Xu [28], which is shown as follows.

**Definition 4 [28]:** Let $\tilde{x}=\left(u_{\tilde{x}},v_{\tilde{x}}\right)$ and $\tilde{y}=\left(u_{\tilde{y}},v_{\tilde{y}}\right)$ be any two IFNs, $S(\tilde{x})$ and $S(\tilde{y})$ be the score functions of $\tilde{x}$ and $\tilde{y}$, and $H(\tilde{x})$ and $H(\tilde{y})$ be the accuracy functions of $\tilde{x}$ and $\tilde{y}$, respectively, then

1. If $S(\tilde{x})>S(\tilde{y})$, then $\tilde{x}>\tilde{y}$;
2. If $S(\tilde{x})=S(\tilde{y})$, then
  If $H(\tilde{x})>H(\tilde{y})$, then $\tilde{x}>\tilde{y}$;
  If $H(\tilde{x})=H(\tilde{y})$, then $\tilde{x}=\tilde{y}$.

### B. MM operator

The MM, which was firstly proposed by Muirhead [42] in 1902, provides a general aggregation function, and it is defined as follows:

**Definition 5 [42].** Let *α*_*i*_ (*i* = 1,2,…,*n*) be a collection of nonnegative real numbers, and *P* = (*p*_1_,*p*_2_,⋯,*p*_*n*_) ∈ *R*^*n*^ be a vector of parameters. If

$$MM^{P}\left(\alpha_{1},\alpha_{2},\ldots ,\alpha_{n}\right)={\left(\frac{1}{n!}\sum_{\vartheta \in S_{n}}\prod_{j=1}^{n}\alpha_{\vartheta (j)}^{p_{j}}\right)}^{\frac{1}{\sum_{j=1}^{n}p_{j}}}$$ (8)

Then we call *MM*^*P*^ the Muirhead mean (MM), where *ϑ*(*j*)(*j* = 1,2,⋯,*n*) is any a permutation of (1,2,⋯,*n*), and *S*_*n*_ is the collection of all permutations of (1,2,⋯,*n*).

In addition, from Eq (8), we can know that

1. If *P* = (1,0,⋯,0), the MM reduces to $MM^{\left(1,0,\cdots ,0\right)}\left(\alpha_{1},\alpha_{2},\ldots ,\alpha_{n}\right)=\frac{1}{n}\sum_{j=1}^{n}\alpha_{j}$ which is the arithmetic averaging operator.
2. If P=(1n,1n,⋯,1n), the MM reduces to MM(1n,1n,⋯,1n)(α1,α2,…,αn)=∏j=1nαj1/n which is the geometric averaging operator.
3. If *P* = (1,1,0,0,⋯,0), the MM reduces to $MM^{\left(1,1,0,0,\cdots ,0\right)}\left(\alpha_{1},\alpha_{2},\ldots ,\alpha_{n}\right)={\left(\frac{1}{n\left(n-1\right)}\sum_{\begin{array}{l} i,j=1\\ i\neq j \end{array}}^{n}\alpha_{i}^{}\alpha_{j}^{}\right)}^{\frac{1}{2}}$ which is the BM operator [36].
4. If $P=\overset{k}{\overset{︷}{(1,1,\cdots ,1}}\overset{n-k}{\overset{︷}{,0,0,\cdots ,0)}}$, the MM reduces to $MM^{\overset{k}{\overset{︷}{(1,1,\cdots ,1}}\overset{n-k}{\overset{︷}{,0,0,\cdots ,0)}}}\left(\alpha_{1},\alpha_{2},\ldots ,\alpha_{n}\right)={\left(\frac{\underset{1\leq i_{1}≺\ldots ≺i_{k}\leq n}{⊕}\overset{k}{\underset{j=1}{⊗}}\alpha_{i_{j}}}{C_{n}^{k}}\right)}^{\frac{1}{k}}$ which is the Maclaurin symmetric mean (MSM) operator [46].

From the definition 5 and the special cases of MM operator mentioned-above, we can know that the advantage of the MM operator is that it can capture the overall interrelationships among the multiple aggregated arguments and it is a generalization of most existing aggregation operators.

## Some Intuitionistic Fuzzy muirhead Mean Operators

Because the traditional MM can only process the crisp number, and IFNs can easily express the fuzzy information, it is necessary to extend MM to process IFNs. In this section, we will propose some intuitionistic fuzzy Muirhead mean operators for the intuitionistic fuzzy information, and discuss some properties of the new operators.

### A. Intuitionistic fuzzy Muirhead mean (IFMM) operator

**Definition 6.** Let *α*_*i*_ = (*u*_*i*_,*v*_*i*_)(*i* = 1,2,…,*n*) be a collection of IFNs, and *P* = (*p*_1_,*p*_2_,⋯,*p*_*n*_) ∈ *R*^*n*^ be a vector of parameters. If

$$IFMM^{P}\left(\alpha_{1},\alpha_{2},\ldots ,\alpha_{n}\right)={\left(\frac{1}{n!}\sum_{\vartheta \in S_{n}}\prod_{j=1}^{n}\alpha_{\vartheta (j)}^{p_{j}}\right)}^{\frac{1}{\sum_{j=1}^{n}p_{j}}}$$ (9)

Then we call *IFMM*^*P*^ the intuitionistic fuzzy MM (IFMM), where *ϑ*(*j*)(*j* = 1,2,⋯,*n*) is any a permutation of (1,2,⋯,*n*), and *S*_*n*_ is the collection of all permutations of (1,2,⋯,*n*).

**Theorem 1.** Let *α*_*i*_ = (*u*_*i*_,*v*_*i*_)(*i* = 1,2,…,*n*) be a collection of the IFNs, then, the aggregation result from Definition 6 is still an IFN, and it can be obtained that

$$IFMM^{P}\left(\alpha_{1},\alpha_{2},\ldots ,\alpha_{n}\right)=\left({\left(1-{\left(\prod_{\vartheta \in S_{n}}\left(1-\prod_{j=1}^{n}u_{\vartheta (j)}^{p_{j}}\right)\right)}^{\frac{1}{n!}}\right)}^{\frac{1}{\sum_{j=1}^{n}p_{j}}},1-{\left(1-{\left(\prod_{\vartheta \in S_{n}}\left(1-\prod_{j=1}^{n}{\left(1-v_{\vartheta (j)}\right)}^{p_{j}}\right)\right)}^{\frac{1}{n!}}\right)}^{\frac{1}{\sum_{j=1}^{n}p_{j}}}\right)$$ (10)

**Proof.** We need to prove (1) Eq (10) is kept; (2) Eq (10) is an IFN.

1. Firstly, we prove Eq (10) is kept.
  According to the operational laws of IFNs, we get
  $$\alpha_{\vartheta (j)}^{p_{j}}=\left(u_{\vartheta (j)}{}^{p_{j}},1-{\left(1-v_{\vartheta (j)}\right)}^{p_{j}}\right),\;and\;\prod_{j=1}^{n}\alpha_{\vartheta (j)}^{p_{j}}=\left(\prod_{j=1}^{n}u_{\vartheta (j)}^{p_{j}},1-\prod_{j=1}^{n}{\left(1-v_{\vartheta (j)}\right)}^{p_{j}}\right),$$
  then
  $$\sum_{\vartheta \in S_{n}}\prod_{j=1}^{n}\alpha_{\vartheta (j)}^{p_{j}}=\left(1-\prod_{\vartheta \in S_{n}}\left(1-\prod_{j=1}^{n}u_{\vartheta (j)}^{p_{j}}\right),\prod_{\vartheta \in S_{n}}\left(1-\prod_{j=1}^{n}{\left(1-v_{\vartheta (j)}\right)}^{p_{j}}\right)\right),$$
  Further,
  $$\frac{1}{n!}\sum_{\vartheta \in S_{n}}\prod_{j=1}^{n}\alpha_{\vartheta (j)}^{p_{j}}=\left(1-{\left(\prod_{\vartheta \in S_{n}}\left(1-\prod_{j=1}^{n}u_{\vartheta (j)}^{p_{j}}\right)\right)}^{\frac{1}{n!}},{\left(\prod_{\vartheta \in S_{n}}\left(1-\prod_{j=1}^{n}{\left(1-v_{\vartheta (j)}\right)}^{p_{j}}\right)\right)}^{\frac{1}{n!}}\right),$$
  So, we have
  $${\left(\frac{1}{n!}\sum_{\vartheta \in S_{n}}\prod_{j=1}^{n}\alpha_{\vartheta (j)}^{p_{j}}\right)}^{\frac{1}{\sum_{j=1}^{n}p_{j}}}=\left({\left(1-{\left(\prod_{\vartheta \in S_{n}}\left(1-\prod_{j=1}^{n}u_{\vartheta (j)}^{p_{j}}\right)\right)}^{\frac{1}{n!}}\right)}^{\frac{1}{\sum_{j=1}^{n}p_{j}}},1-{\left(1-{\left(\prod_{\vartheta \in S_{n}}\left(1-\prod_{j=1}^{n}{\left(1-v_{\vartheta (j)}\right)}^{p_{j}}\right)\right)}^{\frac{1}{n!}}\right)}^{\frac{1}{\sum_{j=1}^{n}p_{j}}}\right).$$
  i.e., Eq (10) is kept.
2. Then we will prove that (10) is an IFN.
  Let
  $$u={\left(1-{\left(\prod_{\vartheta \in S_{n}}\left(1-\prod_{j=1}^{n}u_{\vartheta (j)}^{p_{j}}\right)\right)}^{\frac{1}{n!}}\right)}^{\frac{1}{\sum_{j=1}^{n}p_{j}}},\;v=1-{\left(1-{\left(\prod_{\vartheta \in S_{n}}\left(1-\prod_{j=1}^{n}{\left(1-v_{\vartheta (j)}\right)}^{p_{j}}\right)\right)}^{\frac{1}{n!}}\right)}^{\frac{1}{\sum_{j=1}^{n}p_{j}}}$$
  Then we need to prove the following two conditions.

(i) 0≤*u*≤1,0≤*v*≤1;
(ii) 0≤*u*+*v*≤1.

(i) Since *u*_*ϑ*(*j*)_ ∈ [0,1], we can get
  $$u_{\vartheta (j)}{}^{p_{j}}\in [0,1]\;and\;\prod_{j=1}^{n}u_{\vartheta (j)}^{p_{j}}\in [0,1],$$
  then
  $$1-\prod_{j=1}^{n}u_{\vartheta (j)}^{p_{j}}\in [0,1],\;\prod_{\vartheta \in S_{n}}\left(1-\prod_{j=1}^{n}u_{\vartheta (j)}^{p_{j}}\right)\in [0,1],\;and\;{\left(\prod_{\vartheta \in S_{n}}\left(1-\prod_{j=1}^{n}u_{\vartheta (j)}^{p_{j}}\right)\right)}^{\frac{1}{n!}}\in [0,1].$$
  Further,
  $$1-{\left(\prod_{\vartheta \in S_{n}}\left(1-\prod_{j=1}^{n}u_{\vartheta (j)}^{p_{j}}\right)\right)}^{\frac{1}{n!}}\in [0,1],\;and\;{\left(1-{\left(\prod_{\vartheta \in S_{n}}\left(1-\prod_{j=1}^{n}u_{\vartheta (j)}^{p_{j}}\right)\right)}^{\frac{1}{n!}}\right)}^{\frac{1}{\sum_{j=1}^{n}p_{j}}}\in [0,1].$$
  i.e., 0 ≤ *u* ≤ 1.
  Similarly, we can get 0 ≤ *v* ≤ 1.
  So, condition (i) is met.
(ii) Since *u*_*ϑ*(*j*)_ + *v*_*ϑ*(*j*)_ ≤ 1, then *u*_*ϑ*(*j*)_ ≤ 1 − *v*_*ϑ*(*j*)_, we can get the following inequality
  $$\begin{array}{l} u+v={\left(1-{\left(\prod_{\vartheta \in S_{n}}\left(1-\prod_{j=1}^{n}u_{\vartheta (j)}^{p_{j}}\right)\right)}^{\frac{1}{n!}}\right)}^{\frac{1}{\sum_{j=1}^{n}p_{j}}}+1-{\left(1-{\left(\prod_{\vartheta \in S_{n}}\left(1-\prod_{j=1}^{n}{\left(1-v_{\vartheta (j)}\right)}^{p_{j}}\right)\right)}^{\frac{1}{n!}}\right)}^{\frac{1}{\sum_{j=1}^{n}p_{j}}}\\ \leq {\left(1-{\left(\prod_{\vartheta \in S_{n}}\left(1-\prod_{j=1}^{n}{\left(1-v_{\vartheta (j)}\right)}^{p_{j}}\right)\right)}^{\frac{1}{n!}}\right)}^{\frac{1}{\sum_{j=1}^{n}p_{j}}}+1-{\left(1-{\left(\prod_{\vartheta \in S_{n}}\left(1-\prod_{j=1}^{n}{\left(1-v_{\vartheta (j)}\right)}^{p_{j}}\right)\right)}^{\frac{1}{n!}}\right)}^{\frac{1}{\sum_{j=1}^{n}p_{j}}}=1. \end{array}$$
  i.e., 0 ≤ *u* + *v* ≤ 1.

According to (i) and (ii), we can know the aggregation result from (10) is still an IFN.

**Example 1.** Let (0.5,0.3), (0.7,0,1), and (0.8, 0.2) be three IFNs, and *P* = (1.0,0.5,0.4), then according to (10), we have

$$\begin{array}{l} IFMM^{\left(1.0,0.5,0.4\right)}\left(\left(0.5,0.3\right),\;\left(0.7,0,1\right),(0.8,0.2)\right)=\\ \left({\left(1-{\left(\prod_{\vartheta \in S_{n}}\left(1-\prod_{j=1}^{n}u_{\vartheta (j)}^{p_{j}}\right)\right)}^{\frac{1}{n!}}\right)}^{\frac{1}{\sum_{j=1}^{n}p_{j}}},1-{\left(1-{\left(\prod_{\vartheta \in S_{n}}\left(1-\prod_{j=1}^{n}{\left(1-v_{\vartheta (j)}\right)}^{p_{j}}\right)\right)}^{\frac{1}{n!}}\right)}^{\frac{1}{\sum_{j=1}^{n}p_{j}}}\right)\\ ((1-(\left(1-0.5^{1.0}\times 0.7^{0.5}\times 0.8^{0.4})\times (1-0.5^{1.0}\times 0.8^{0.5}\times 0.7^{0.4})\times (1-0.7^{1.0}\times 0.5^{0.5}\times 0.8^{0.4}\right)\\ {{\times (1-0.7^{1.0}\times 0.8^{0.5}\times 0.5^{0.4})\times (1-0.8^{1.0}\times 0.5^{0.5}\times 0.7^{0.4})\times (1-0.8^{1.0}\times 0.7^{0.5}\times 0.5^{0.4}))}^{\frac{1}{3!}})}^{\frac{1}{1.0+0.5+0.4}},\\ 1-(1-(\left(1-{\left(1-0.3\right)}^{1.0}\times {\left(1-0.1\right)}^{0.5}\times {\left(1-0.2\right)}^{0.4}\right)\times \left(1-{\left(1-0.3\right)}^{1.0}\times {\left(1-0.2\right)}^{0.5}\times {\left(1-0.1\right)}^{0.4}\right)\times \\ \left(1-{\left(1-0.1\right)}^{1.0}\times {\left(1-0.3\right)}^{0.5}\times {\left(1-0.2\right)}^{0.4}\right)\times \left(1-{\left(1-0.1\right)}^{1.0}\times {\left(1-0.2\right)}^{0.5}\times {\left(1-0.3\right)}^{0.4}\right)\times \\ {{\left(1-{\left(1-0.2\right)}^{1.0}\times {\left(1-0.3\right)}^{0.5}\times {\left(1-0.1\right)}^{0.4}\right)\times \left(1-{\left(1-0.2\right)}^{1.0}\times {\left(1-0.1\right)}^{0.5}\times {\left(1-0.3\right)}^{0.4}\right))}^{\frac{1}{3!}})}^{\frac{1}{1.0+0.5+0.4}})\\ =\left(0.658,0.202\right). \end{array}$$

Next, we will discuss some properties of IFMM operator.

**Property 1 (Idempotency).** If all *α*_*i*_ (*i* = 1,2,…,*n*) are equal, i.e., *α*_*i*_ = *α* = (*u*,*v*), then

$$IFMM^{P}\left(\alpha_{1},\alpha_{2},\ldots ,\alpha_{n}\right)=\alpha .$$

**Proof.** Since *α*_*i*_ = *α* = (*u*,*v*), based on Theorem 1, we get

$$\begin{array}{l} IFMM^{P}\left(\alpha ,\alpha ,\ldots ,\alpha \right)=\left({\left(1-{\left(\prod_{\vartheta \in S_{n}}\left(1-\prod_{j=1}^{n}u_{}^{p_{j}}\right)\right)}^{\frac{1}{n!}}\right)}^{\frac{1}{\sum_{j=1}^{n}p_{j}}},1-{\left(1-{\left(\prod_{\vartheta \in S_{n}}\left(1-\prod_{j=1}^{n}{\left(1-v\right)}^{p_{j}}\right)\right)}^{\frac{1}{n!}}\right)}^{\frac{1}{\sum_{j=1}^{n}p_{j}}}\right)\\ =\left({\left(1-{\left(\prod_{\vartheta \in S_{n}}\left(1-u_{}^{\sum_{j=1}^{n}p_{j}}\right)\right)}^{\frac{1}{n!}}\right)}^{\frac{1}{\sum_{j=1}^{n}p_{j}}},1-{\left(1-{\left(\prod_{\vartheta \in S_{n}}\left(1-{\left(1-v\right)}^{\sum_{j=1}^{n}p_{j}}\right)\right)}^{\frac{1}{n!}}\right)}^{\frac{1}{\sum_{j=1}^{n}p_{j}}}\right)\\ =\left({\left(1-{\left({\left(1-u_{}^{\sum_{j=1}^{n}p_{j}}\right)}^{n!}\right)}^{\frac{1}{n!}}\right)}^{\frac{1}{\sum_{j=1}^{n}p_{j}}},1-{\left(1-{\left({\left(1-{\left(1-v\right)}^{\sum_{j=1}^{n}p_{j}}\right)}^{n!}\right)}^{\frac{1}{n!}}\right)}^{\frac{1}{\sum_{j=1}^{n}p_{j}}}\right)\\ =\left({\left(1-\left(1-u_{}^{\sum_{j=1}^{n}p_{j}}\right)\right)}^{\frac{1}{\sum_{j=1}^{n}p_{j}}},1-{\left(1-\left(1-{\left(1-v\right)}^{\sum_{j=1}^{n}p_{j}}\right)\right)}^{\frac{1}{\sum_{j=1}^{n}p_{j}}}\right)\\ =\left({\left(u_{}^{\sum_{j=1}^{n}p_{j}}\right)}^{\frac{1}{\sum_{j=1}^{n}p_{j}}},1-{\left({\left(1-v\right)}^{\sum_{j=1}^{n}p_{j}}\right)}^{\frac{1}{\sum_{j=1}^{n}p_{j}}}\right)=\left(u,1-(1-v)\right)=(u,v). \end{array}$$

**Property 2 (Monotonicity).** Let *α*_*i*_ = (*u*_*i*_,*v*_*i*_) and $\alpha_{i}^{\prime }=\left(u_{i}^{\prime },v_{i}^{\prime }\right)$ (*i* = 1,2,…,*n*) be two sets of IFNs. If $u_{i}\geq u_{i}^{\prime },v_{i}\leq v_{i}^{\prime }$ for all *i*, then

$$IFMM^{P}\left(\alpha_{1},\alpha_{2},\ldots ,\alpha_{n}\right)\geq IFMM^{P}\left(\alpha_{1}^{\prime },\alpha_{2}^{\prime },\ldots ,\alpha_{n}^{\prime }\right).$$

**Proof.** Let $IFMM^{P}\left(\alpha_{1},\alpha_{2},\ldots ,\alpha_{n}\right)=(u,v),IFMM^{P}\left(\alpha_{1}^{\prime },\alpha_{2}^{\prime },\ldots ,\alpha_{n}^{\prime }\right)=(u^{\prime },v^{\prime })$, where

$$u={\left(1-{\left(\prod_{\vartheta \in S_{n}}\left(1-\prod_{j=1}^{n}u_{\vartheta (j)}^{p_{j}}\right)\right)}^{\frac{1}{n!}}\right)}^{\frac{1}{\sum_{j=1}^{n}p_{j}}},u^{\prime }={\left(1-{\left(\prod_{\vartheta \in S_{n}}\left(1-\prod_{j=1}^{n}u_{\vartheta (j)}^{\prime p_{j}}\right)\right)}^{\frac{1}{n!}}\right)}^{\frac{1}{\sum_{j=1}^{n}p_{j}}},$$

and

$$v=1-{\left(1-{\left(\prod_{\vartheta \in S_{n}}\left(1-\prod_{j=1}^{n}{\left(1-v_{\vartheta (j)}\right)}^{p_{j}}\right)\right)}^{\frac{1}{n!}}\right)}^{\frac{1}{\sum_{j=1}^{n}p_{j}}},v^{\prime }=1-{\left(1-{\left(\prod_{\vartheta \in S_{n}}\left(1-\prod_{j=1}^{n}{\left(1-v_{\vartheta (j)}^{\prime }\right)}^{p_{j}}\right)\right)}^{\frac{1}{n!}}\right)}^{\frac{1}{\sum_{j=1}^{n}p_{j}}}$$

Since $u_{i}\geq u_{i}^{\prime }$, we can get

$$u_{\vartheta (j)}{}^{p_{j}}\geq u_{\vartheta (j)}^{\prime }{}^{p_{j}}\;and\;\prod_{j=1}^{n}u_{\vartheta (j)}^{p_{j}}\geq \prod_{j=1}^{n}u_{\vartheta (j)}^{\prime p_{j}},$$

then

$$1-\prod_{j=1}^{n}u_{\vartheta (j)}^{p_{j}}\leq 1-\prod_{j=1}^{n}u_{\vartheta (j)}^{\prime p_{j}},\;\prod_{\vartheta \in S_{n}}\left(1-\prod_{j=1}^{n}u_{\vartheta (j)}^{p_{j}}\right)\leq \prod_{\vartheta \in S_{n}}\left(1-\prod_{j=1}^{n}u_{\vartheta (j)}^{\prime p_{j}}\right),$$

and

$${\left(\prod_{\vartheta \in S_{n}}\left(1-\prod_{j=1}^{n}u_{\vartheta (j)}^{p_{j}}\right)\right)}^{\frac{1}{n!}}\leq {\left(\prod_{\vartheta \in S_{n}}\left(1-\prod_{j=1}^{n}u_{\vartheta (j)}^{\prime p_{j}}\right)\right)}^{\frac{1}{n!}}.$$

Further,

$$1-{\left(\prod_{\vartheta \in S_{n}}\left(1-\prod_{j=1}^{n}u_{\vartheta (j)}^{p_{j}}\right)\right)}^{\frac{1}{n!}}\geq 1-{\left(\prod_{\vartheta \in S_{n}}\left(1-\prod_{j=1}^{n}u_{\vartheta (j)}^{\prime p_{j}}\right)\right)}^{\frac{1}{n!}},$$

and

$${\left(1-{\left(\prod_{\vartheta \in S_{n}}\left(1-\prod_{j=1}^{n}u_{\vartheta (j)}^{p_{j}}\right)\right)}^{\frac{1}{n!}}\right)}^{\frac{1}{\sum_{j=1}^{n}p_{j}}}\geq {\left(1-{\left(\prod_{\vartheta \in S_{n}}\left(1-\prod_{j=1}^{n}u_{\vartheta (j)}^{\prime p_{j}}\right)\right)}^{\frac{1}{n!}}\right)}^{\frac{1}{\sum_{j=1}^{n}p_{j}}}.$$

i.e., *u* ≥ *u*′.

Similarly, we also have *v* ≤ *v*′.

In the following, we will discuss three situations as follows.

If *u* > *u*′, because *v* ≤ *v*′, then $IFMM^{P}\left(\alpha_{1},\alpha_{2},\ldots ,\alpha_{n}\right)>FMM^{P}\left(\alpha_{1}^{\prime },\alpha_{2}^{\prime },\ldots ,\alpha_{n}^{\prime }\right)$.

If *u* = *u*′ and *v* < *v*′, then $IFMM^{P}\left(\alpha_{1},\alpha_{2},\ldots ,\alpha_{n}\right)>IFMM^{P}\left(\alpha_{1}^{\prime },\alpha_{2}^{\prime },\ldots ,\alpha_{n}^{\prime }\right)$

If *u* = *u*′ and *v* = *v*′, then $IFMM^{P}\left(\alpha_{1},\alpha_{2},\ldots ,\alpha_{n}\right)=IFMM^{P}\left(\alpha_{1}^{\prime },\alpha_{2}^{\prime },\ldots ,\alpha_{n}^{\prime }\right)$

So, Property 2 is kept.

**Property 3 (Boundedness).** Let *α*_*i*_ = (*u*_*i*_,*v*_*i*_) (*i* = 1,2,…,*n*) be a collections of IFNs, and *α*^−^ = (min(*u*_*i*_),max(*v*_*i*_)), *α*^+^ = (max(*u*_*i*_),min(*v*_*i*_)), Then *α*^−^ ≤ *IFMM*^*P*^ (*α*_1_,*α*_2_,…,*α*_*n*_) ≤ *α*^+^.

**Proof.** According to Properties 1 and 2, we have

$$IFMM^{P}\left(\alpha_{1},\alpha_{2},\ldots ,\alpha_{n}\right)\geq IFMM^{P}\left(\alpha^{-},\alpha^{-},\ldots ,\alpha^{-}\right)=\alpha^{-}$$

and

$$IFMM^{P}\left(\alpha_{1},\alpha_{2},\ldots ,\alpha_{n}\right)\leq IFMM^{P}\left(\alpha^{+},\alpha^{+},\ldots ,\alpha^{+}\right)=\alpha^{+}.$$

So, we have

$$\alpha^{-}\leq IFMM^{P}\left(\alpha_{1},\alpha_{2},\ldots ,\alpha_{n}\right)\leq \alpha^{+}.$$

In the following, we will explore some special cases of IFMM operator with respect to the parameter vector.

1. If *P* = (1,0,⋯,0), the IFMM reduces to the intuitionistic fuzzy arithmetic averaging operator [27].
  $$IFMM^{\left(1,0,\cdots ,0\right)}\left(\alpha_{1},\alpha_{2},\ldots ,\alpha_{n}\right)=\frac{1}{n}\sum_{i=1}^{n}\alpha_{i}=\left(1-\prod_{j=1}^{n}{\left(1-u_{i}\right)}^{1/n},\prod_{j=1}^{n}v_{i}^{1/n}\right)$$ (11)
2. If *P* = (*λ*,0,⋯,0), the IFMM reduces to the intuitionistic fuzzy generalized arithmetic averaging operator [47].
  $$IFMM^{\left(\lambda ,0,\cdots ,0\right)}\left(\alpha_{1},\alpha_{2},\ldots ,\alpha_{n}\right)={\left(\frac{1}{n}\sum_{i=1}^{n}\alpha_{i}^{\lambda }\right)}^{1/\lambda }=\left({\left(1-\prod_{j=1}^{n}{\left(1-u_{i}{}^{\lambda }\right)}^{1/n}\right)}^{1/\lambda },1-{\left(1-{\prod_{j=1}^{n}\left(1-{\left(1-v_{i}\right)}^{\lambda }\right)}^{1/n}\right)}^{1/\lambda }\right)$$ (12)
3. If *P* = (1,1,0,0,⋯,0), the IFMM reduces to the intuitionistic fuzzy BM operator [48].
  $$\begin{array}{l} IFMM^{\left(1,1,0,0,\cdots ,0\right)}\left(\alpha_{1},\alpha_{2},\ldots ,\alpha_{n}\right)={\left(\frac{1}{n\left(n-1\right)}\sum_{\begin{array}{l} i,j=1\\ i\neq j \end{array}}^{n}\alpha_{i}^{}\alpha_{j}^{}\right)}^{\frac{1}{2}}\\ =\left({\left(1-{\prod_{\begin{array}{l} i,j=1\\ i\neq j \end{array}}^{n}\left(1-u_{i}u_{j}\right)}^{\frac{1}{n\left(n-1\right)}}\right)}^{1/2},1-{\left(1-{\prod_{\begin{array}{l} i,j=1\\ i\neq j \end{array}}^{n}\left(v_{i}+v_{j}-v_{i}v_{j}\right)}^{\frac{1}{n\left(n-1\right)}}\right)}^{1/2}\right) \end{array}$$ (13)
4. If $P=\overset{k}{\overset{︷}{(1,1,\cdots ,1}}\overset{n-k}{\overset{︷}{,0,0,\cdots ,0)}}$, the IFMM reduces to the intuitionistic fuzzy Maclaurin symmetric mean (MSM) operator [43].
  $$\begin{array}{l} IFMM^{\overset{k}{\overset{︷}{(1,1,\cdots ,1}}\overset{n-k}{\overset{︷}{,0,0,\cdots ,0)}}}\left(\alpha_{1},\alpha_{2},\ldots ,\alpha_{n}\right)={\left(\frac{\underset{1\leq i_{1}≺\ldots ≺i_{k}\leq n}{⊕}\overset{k}{\underset{j=1}{⊗}}\alpha_{i_{j}}}{C_{n}^{k}}\right)}^{\frac{1}{k}}\\ =\left({\left(1-{\prod_{1\leq i_{1}≺\ldots ≺i_{k}\leq n}^{}\left(1-\prod_{j=1}^{k}u_{i_{j}}\right)}^{1/C_{n}^{k}}\right)}^{1/k},1-{\left(1-{\prod_{1\leq i_{1}≺\ldots ≺i_{k}\leq n}^{}\left(1-\prod_{j=1}^{k}\left(1-v_{i_{j}}\right)\right)}^{1/C_{n}^{k}}\right)}^{1/k}\right) \end{array}$$ (14)
5. If *P* = (1,1,⋯,1), the IFMM reduces to the intuitionistic fuzzy geometric averaging operator [28].
  $$IFMM^{\left(1,1,\cdots ,1\right)}\left(\alpha_{1},\alpha_{2},\ldots ,\alpha_{n}\right)={\left(\prod_{j=1}^{n}\alpha_{j}^{}\right)}^{1/n}=\left(\prod_{j=1}^{n}u_{j}^{1/n},1-{\left(\prod_{j=1}^{n}\left(1-v_{j}^{}\right)\right)}^{1/n}\right)$$ (15)
6. If P=(1n,1n,⋯,1n), the IFMM reduces to the intuitionistic fuzzy geometric averaging operator [28].
  IFMM(1n,1n,⋯,1n)(α1,α2,…,αn)=∏j=1nαj1/n=(∏j=1nuj1/n,1−(∏j=1n(1−vj))1/n) (16)

Further, in order to discuss the monotonic of IFMM operator about the parameter vector *P* ∈ *R*^*n*^, we firstly cited a lemma.

**Lemma 1 [49].** Let *P* = (*p*_1_,*p*_2_,⋯,*p*_*n*_) and *Q* = (*q*_1_,*q*_2_,⋯,*q*_*n*_) be two the parameter vectors, if

$$\sum_{j=1}^{k}p_{\left[j\right]}\leq \sum_{j=1}^{k}q_{\left[j\right]}\;(j=1,2,\cdots ,n-1)$$

$$\sum_{j=1}^{n}p_{j}=\sum_{j=1}^{n}q_{j}$$ (17)

Where ([1],[2],⋯,[*n*]) is a permutation of (*i* = 1,2,…,*n*) and meets *p*_[*j*]_ ≥ *p*_[*j*+1]_,*q*_[*j*]_ ≥ *q*_[*j*+1]_ for all (*i* = 1,2,…,*n*). Then it can call that *P* is controlled by vector *Q*, expressed by *P* ≺ *Q*.

**Theorem 2**. Let *α*_*i*_ = (*u*_*i*_,*v*_*i*_) (*i* = 1,2,…,*n*) be a collections of IFNs, and *P* = (*p*_1_,*p*_2_,⋯,*p*_*n*_), *Q* = (*q*_1_,*q*_2_,⋯,*q*_*n*_) be two the parameter vectors, if *P* ≺ *Q*, then

$$IFMM^{P}\left(\alpha_{1},\alpha_{2},\ldots ,\alpha_{n}\right)\leq IFMM^{Q}\left(\alpha_{1},\alpha_{2},\ldots ,\alpha_{n}\right)$$ (18)

The proof this theorem is omitted, please refer to [44].

### B. The intuitionistic fuzzy weighted MM operator

In actual decision making, the weights of attributes will directly influence the decision-making results. However, IFMM operator cannot consider the attribute weights, so it is very important to take into account the weights of attributes for information aggregation. In this subsection, we will propose a weighted IFMM operator as follows.

**Definition 7.** Let *α*_*i*_ = (*u*_*i*_,*v*_*i*_)(*i* = 1,2,…,*n*) be a collection of IFNs, *w* = (*w*_1_,*w*_2_,…,*w*_*n*_)^*T*^ be the weight vector of *α*_*i*_(*i* = 1,2,…,*n*), which satisfies *w*_*i*_ ∈ [0,1] and $\sum_{i=1}^{n}w_{i}=1$, and let *P* = (*p*_1_,*p*_2_,⋯,*p*_*n*_) ∈ *R*^*n*^ be a vector of parameters. If

$$IFWMM^{P}\left(\alpha_{1},\alpha_{2},\ldots ,\alpha_{n}\right)={\left(\frac{1}{n!}\sum_{\vartheta \in S_{n}}\prod_{j=1}^{n}{\left(nw_{\vartheta (j)}\alpha_{\vartheta (j)}\right)}^{p_{j}}\right)}^{\frac{1}{\sum_{j=1}^{n}p_{j}}}$$ (19)

Then we call *IFWMM*^*P*^ the intuitionistic fuzzy weighted MM (IFWMM), where *ϑ*(*j*)(*j* = 1,2,⋯,*n*) is any a permutation of (1,2,⋯,*n*), and *S*_*n*_ is the collection of all permutations of (1,2,⋯,*n*).

**Theorem 3**. Let *α*_*i*_ = (*u*_*i*_,*v*_*i*_)(*i* = 1,2,…,*n*) be a collection of IFNs, then the result from Definition 7 is an IFN, and it can be obtained that

$$IFWMM^{P}\left(\alpha_{1},\alpha_{2},\ldots ,\alpha_{n}\right)=\left({\left(1-{\left(\prod_{\vartheta \in S_{n}}\left(1-\prod_{j=1}^{n}{\left(1-{\left(1-u_{\vartheta (j)}\right)}^{nw_{\vartheta (j)}}\right)}^{p_{j}}\right)\right)}^{\frac{1}{n!}}\right)}^{\frac{1}{\sum_{j=1}^{n}p_{j}}},1-{\left(1-{\left(\prod_{\vartheta \in S_{n}}\left(1-\prod_{j=1}^{n}{\left(1-v_{\vartheta (j)}^{nw_{\vartheta (j)}}\right)}^{p_{j}}\right)\right)}^{\frac{1}{n!}}\right)}^{\frac{1}{\sum_{j=1}^{n}p_{j}}}\right)$$ (20)

**Proof.** Because $nw_{\vartheta (j)}\alpha_{\vartheta (j)}=\left(1-{\left(1-u_{\vartheta (j)}\right)}^{nw_{\vartheta (j)}},v_{\vartheta (j)}^{nw_{\vartheta (j)}}\right)$, we can replace *u*_*ϑ*(*j*)_ in Eq (10) with $1-{\left(1-u_{\vartheta (j)}\right)}^{nw_{\vartheta (j)}}$, and *v*_*ϑ*(*j*)_ in Eq (10) with $v_{\vartheta (j)}^{nw_{\vartheta (j)}}$, then we can get Eq (20).

Because *α*_*ϑ*(*j*)_ is an IFN, *nw*_*ϑ*(*j*)_*α*_*ϑ*(*j*)_ is also an IFN. By Eq (10), we know *IFWMM*^*P*^ (*α*_1_,*α*_2_,…,*α*_*n*_) is an IFN.

In the following, we shall explore some desirable properties of IFWMM operator.

**Property 4 (Monotonicity).** Let *α*_*i*_ = (*u*_*i*_,*v*_*i*_) and $\alpha_{i}^{\prime }=\left(u_{i}^{\prime },v_{i}^{\prime }\right)$ (*i* = 1,2,…,*n*) be two sets of IFNs. If $u_{i}\geq u_{i}^{\prime },v_{i}\leq v_{i}^{\prime }$ for all *i*, then

$$IFWMM^{P}\left(\alpha_{1},\alpha_{2},\ldots ,\alpha_{n}\right)\geq IFWMM^{P}\left(\alpha_{1}^{\prime },\alpha_{2}^{\prime },\ldots ,\alpha_{n}^{\prime }\right).$$

The Proof is similar to that of IFMM operator, it is omitted here.

**Property 5 (Boundedness).** Let *α*_*i*_ = (*u*_*i*_,*v*_*i*_) (*i* = 1,2,…,*n*) be a collections of IFNs, and *α*^−^ = (min(*u*_*i*_),max(*v*_*i*_)), *α*^+^ = (max(*u*_*i*_),min(*v*_*i*_)), then $\left(u_{\alpha^{-}},v_{\alpha^{-}}\right)\leq IFWMM^{P}\left(\alpha_{1},\alpha_{2},\ldots ,\alpha_{n}\right)\leq \left(u_{\alpha^{+}},v_{\alpha^{+}}\right)$.

Where,

$$u_{\alpha^{-}}={\left(1-{\left(\prod_{\vartheta \in S_{n}}\left(1-\prod_{j=1}^{n}{\left(1-{\left(1-\min(u_{i})\right)}^{nw_{\vartheta (j)}}\right)}^{p_{j}}\right)\right)}^{\frac{1}{n!}}\right)}^{\frac{1}{\sum_{j=1}^{n}p_{j}}},\;v_{\alpha^{-}}=1-{\left(1-{\left(\prod_{\vartheta \in S_{n}}\left(1-\prod_{j=1}^{n}{\left(1-\max{\left(v_{i}\right)}_{}^{nw_{\vartheta (j)}}\right)}^{p_{j}}\right)\right)}^{\frac{1}{n!}}\right)}^{\frac{1}{\sum_{j=1}^{n}p_{j}}},$$

$$u_{\alpha^{+}}={\left(1-{\left(\prod_{\vartheta \in S_{n}}\left(1-\prod_{j=1}^{n}{\left(1-{\left(1-\max(u_{i})\right)}^{nw_{\vartheta (j)}}\right)}^{p_{j}}\right)\right)}^{\frac{1}{n!}}\right)}^{\frac{1}{\sum_{j=1}^{n}p_{j}}},\;v_{\alpha^{+}}=1-{\left(1-{\left(\prod_{\vartheta \in S_{n}}\left(1-\prod_{j=1}^{n}{\left(1-\min{\left(v_{i}\right)}_{}^{nw_{\vartheta (j)}}\right)}^{p_{j}}\right)\right)}^{\frac{1}{n!}}\right)}^{\frac{1}{\sum_{j=1}^{n}p_{j}}}.$$

**Proof.** According to Property 4, we have

$$IFWMM^{P}\left(\alpha^{-},\alpha^{-},\ldots ,\alpha^{-}\right)\leq IFWMM^{P}\left(\alpha_{1},\alpha_{2},\ldots ,\alpha_{n}\right)\leq IFWMM^{P}\left(\alpha^{+},\alpha^{+},\ldots ,\alpha^{+}\right),$$

According to Eq (20), we have

$$\begin{array}{l} IFWMM^{P}\left(\alpha^{-},\alpha^{-},\ldots ,\alpha^{-}\right)=\\ \left({\left(1-{\left(\prod_{\vartheta \in S_{n}}\left(1-\prod_{j=1}^{n}{\left(1-{\left(1-\min(u_{i})\right)}^{nw_{\vartheta (j)}}\right)}^{p_{j}}\right)\right)}^{\frac{1}{n!}}\right)}^{\frac{1}{\sum_{j=1}^{n}p_{j}}},1-{\left(1-{\left(\prod_{\vartheta \in S_{n}}\left(1-\prod_{j=1}^{n}{\left(1-\max{\left(v_{i}\right)}_{}^{nw_{\vartheta (j)}}\right)}^{p_{j}}\right)\right)}^{\frac{1}{n!}}\right)}^{\frac{1}{\sum_{j=1}^{n}p_{j}}}\right), \end{array}$$

and

$$\begin{array}{l} IFWMM^{P}\left(\alpha^{+},\alpha^{+},\ldots ,\alpha^{+}\right)=\\ \left({\left(1-{\left(\prod_{\vartheta \in S_{n}}\left(1-\prod_{j=1}^{n}{\left(1-{\left(1-\max(u_{i})\right)}^{nw_{\vartheta (j)}}\right)}^{p_{j}}\right)\right)}^{\frac{1}{n!}}\right)}^{\frac{1}{\sum_{j=1}^{n}p_{j}}},1-{\left(1-{\left(\prod_{\vartheta \in S_{n}}\left(1-\prod_{j=1}^{n}{\left(1-\min{\left(v_{i}\right)}_{}^{nw_{\vartheta (j)}}\right)}^{p_{j}}\right)\right)}^{\frac{1}{n!}}\right)}^{\frac{1}{\sum_{j=1}^{n}p_{j}}}\right). \end{array}$$

So,

$$\left(u_{\alpha^{-}},v_{\alpha^{-}}\right)\leq IFMM^{P}\left(\alpha_{1},\alpha_{2},\ldots ,\alpha_{n}\right)\leq \left(u_{\alpha^{+}},v_{\alpha^{+}}\right).$$

Obviously, the IFWMM operator doesn’t have the property of idempotency.

**Theorem 4.** The IFMM operator is a special case of the IFWMM operator.

**Proof.** When $w=\left(\frac{1}{n},\frac{1}{n},\cdots ,\frac{1}{n}\right)$

$$\begin{array}{l} IFWMM^{P}\left(\alpha_{1},\alpha_{2},\ldots ,\alpha_{n}\right)=\left({\left(1-{\left(\prod_{\vartheta \in S_{n}}\left(1-\prod_{j=1}^{n}{\left(1-{\left(1-u_{\vartheta (j)}\right)}^{nw_{\vartheta (j)}}\right)}^{p_{j}}\right)\right)}^{\frac{1}{n!}}\right)}^{\frac{1}{\sum_{j=1}^{n}p_{j}}},1-{\left(1-{\left(\prod_{\vartheta \in S_{n}}\left(1-\prod_{j=1}^{n}{\left(1-v_{\vartheta (j)}^{nw_{\vartheta (j)}}\right)}^{p_{j}}\right)\right)}^{\frac{1}{n!}}\right)}^{\frac{1}{\sum_{j=1}^{n}p_{j}}}\right)\\ =\left({\left(1-{\left(\prod_{\vartheta \in S_{n}}\left(1-\prod_{j=1}^{n}{\left(1-{\left(1-u_{\vartheta (j)}\right)}^{n\times \frac{1}{n}}\right)}^{p_{j}}\right)\right)}^{\frac{1}{n!}}\right)}^{\frac{1}{\sum_{j=1}^{n}p_{j}}},1-{\left(1-{\left(\prod_{\vartheta \in S_{n}}\left(1-\prod_{j=1}^{n}{\left(1-v_{\vartheta (j)}^{n\times \frac{1}{n}}\right)}^{p_{j}}\right)\right)}^{\frac{1}{n!}}\right)}^{\frac{1}{\sum_{j=1}^{n}p_{j}}}\right)\\ =\left({\left(1-{\left(\prod_{\vartheta \in S_{n}}\left(1-\prod_{j=1}^{n}u_{\vartheta (j)}{}^{p_{j}}\right)\right)}^{\frac{1}{n!}}\right)}^{\frac{1}{\sum_{j=1}^{n}p_{j}}},1-{\left(1-{\left(\prod_{\vartheta \in S_{n}}\left(1-\prod_{j=1}^{n}{\left(1-v_{\vartheta (j)}^{}\right)}^{p_{j}}\right)\right)}^{\frac{1}{n!}}\right)}^{\frac{1}{\sum_{j=1}^{n}p_{j}}}\right)\\ =IFMM^{P}\left(\alpha_{1},\alpha_{2},\ldots ,\alpha_{n}\right). \end{array}.$$

### C. The intuitionistic fuzzy dual MM operator

In the theory of aggregation operator, there exist two types, i.e., original operator and its dual operator, for example, arithmetic average operator and geometric average operator. In this section, we will propose the dual MM operator for intuitionistic fuzzy numbers based on the IFMM operator as follows.

**Definition 8.** Let *α*_*i*_ = (*u*_*i*_,*v*_*i*_)(*i* = 1,2,…,*n*) be a collection of IFNs, and let *P* = (*p*_1_,*p*_2_,⋯,*p*_*n*_) ∈ *R*^*n*^ be a vector of parameters. If

$$IFDMM^{P}\left(\alpha_{1},\alpha_{2},\ldots ,\alpha_{n}\right)=\frac{1}{\sum_{j=1}^{n}p_{j}}{\left(\prod_{\vartheta \in S_{n}}\sum_{j=1}^{n}\left(p_{j}\alpha_{\vartheta (j)}\right)\right)}^{\frac{1}{n!}}.$$ (21)

Then we call *IFDMM*^*P*^ the intuitionistic fuzzy dual MM (IFDMM), where *ϑ*(*j*)(*j* = 1,2,⋯,*n*) is any a permutation of (1,2,⋯,*n*), and *S*_*n*_ is the collection of all permutations of (1,2,⋯,*n*).

**Theorem 5**. Let *α*_*i*_ = (*u*_*i*_,*v*_*i*_)(*i* = 1,2,…,*n*) be a collection of IFNs, then, the result from Definition 8 is also an IFN, and it can be obtained that

$$IFDMM^{P}\left(\alpha_{1},\alpha_{2},\ldots ,\alpha_{n}\right)=\left(1-{\left(1-\prod_{\vartheta \in S_{n}}{\left(1-\prod_{j=1}^{n}{\left(1-u_{\vartheta (j)}\right)}^{p_{j}}\right)}^{\frac{1}{n!}}\right)}^{\frac{1}{\sum_{j=1}^{n}p_{j}}},{\left(1-{\left(\prod_{\vartheta \in S_{n}}\left(1-\prod_{j=1}^{n}v_{\vartheta (j)}^{p_{j}}\right)\right)}^{\frac{1}{n!}}\right)}^{\frac{1}{\sum_{j=1}^{n}p_{j}}}\right).$$ (22)

**Proof.** We need to prove (1) Eq (22) is kept; (2) Eq (22) is an IFN.

1. Firstly, we prove the Eq (22) is kept.
  According to the operational laws of IFNs, we get
  $$p_{j}\alpha_{\vartheta (j)}=\left(1-{\left(1-u_{\vartheta (j)}\right)}^{p_{j}},v_{\vartheta (j)}^{p_{j}}\right),\;and\;\sum_{j=1}^{n}\left(p_{j}\alpha_{\vartheta (j)}\right)=\left(1-\prod_{j=1}^{n}{\left(1-u_{\vartheta (j)}\right)}^{p_{j}},\prod_{j=1}^{n}v_{\vartheta (j)}^{p_{j}}\right),$$
  then
  $$\prod_{\vartheta \in S_{n}}\sum_{j=1}^{n}\left(p_{j}\alpha_{\vartheta (j)}\right)=\left(\prod_{\vartheta \in S_{n}}\left(1-\prod_{j=1}^{n}{\left(1-u_{\vartheta (j)}\right)}^{p_{j}}\right),1-\prod_{\vartheta \in S_{n}}\left(1-\prod_{j=1}^{n}v_{\vartheta (j)}^{p_{j}}\right)\right),$$
  Further,
  $${\left(\prod_{\vartheta \in S_{n}}\sum_{j=1}^{n}\left(p_{j}\alpha_{\vartheta (j)}\right)\right)}^{\frac{1}{n!}}=\left(\prod_{\vartheta \in S_{n}}{\left(1-\prod_{j=1}^{n}{\left(1-u_{\vartheta (j)}\right)}^{p_{j}}\right)}^{\frac{1}{n!}},1-{\left(\prod_{\vartheta \in S_{n}}\left(1-\prod_{j=1}^{n}v_{\vartheta (j)}^{p_{j}}\right)\right)}^{\frac{1}{n!}}\right),$$
  So,
  $$\frac{1}{\sum_{j=1}^{n}p_{j}}{\left(\prod_{\vartheta \in S_{n}}\sum_{j=1}^{n}\left(p_{j}\alpha_{\vartheta (j)}\right)\right)}^{\frac{1}{n!}}=\left(1-{\left(1-\prod_{\vartheta \in S_{n}}{\left(1-\prod_{j=1}^{n}{\left(1-u_{\vartheta (j)}\right)}^{p_{j}}\right)}^{\frac{1}{n!}}\right)}^{\frac{1}{\sum_{j=1}^{n}p_{j}}},{\left(1-{\left(\prod_{\vartheta \in S_{n}}\left(1-\prod_{j=1}^{n}v_{\vartheta (j)}^{p_{j}}\right)\right)}^{\frac{1}{n!}}\right)}^{\frac{1}{\sum_{j=1}^{n}p_{j}}}\right).$$
  i.e., Eq (22) is kept.
2. Then we will prove that (22) is an IFN.
  Let
  $$u=1-{\left(1-\prod_{\vartheta \in S_{n}}{\left(1-\prod_{j=1}^{n}{\left(1-u_{\vartheta (j)}\right)}^{p_{j}}\right)}^{\frac{1}{n!}}\right)}^{\frac{1}{\sum_{j=1}^{n}p_{j}}},\;v={\left(1-{\left(\prod_{\vartheta \in S_{n}}\left(1-\prod_{j=1}^{n}v_{\vartheta (j)}^{p_{j}}\right)\right)}^{\frac{1}{n!}}\right)}^{\frac{1}{\sum_{j=1}^{n}p_{j}}}$$
  Then we need prove the following two conditions.

(i) 0 ≤ *u* ≤ 1,0 ≤ *v* ≤ 1;
(ii) 0 ≤ *u* + *v* ≤ 1.
(i) Since *u*_*ϑ*(*j*)_ ∈ [0,1], we can get
  $$\left(1-u_{\vartheta (j)}\right)\in [0,1],\;{\left(1-u_{\vartheta (j)}\right)}^{p_{j}}\in [0,1]\;and\;\prod_{j}^{n}{\left(1-u_{\vartheta (j)}\right)}^{p_{j}}\in [0,1],$$
  then
  $$\left(1-\prod_{j=1}^{n}{\left(1-u_{\vartheta (j)}\right)}^{p_{j}}\right)\in [0,1],\;{\left(1-\prod_{j=1}^{n}{\left(1-u_{\vartheta (j)}\right)}^{p_{j}}\right)}^{\frac{1}{n!}}\in [0,1],\;and\;\prod_{\vartheta \in S_{n}}{\left(1-\prod_{j=1}^{n}{\left(1-u_{\vartheta (j)}\right)}^{p_{j}}\right)}^{\frac{1}{n!}}\in [0,1].$$
  Further,
  $$\left(1-\prod_{\vartheta \in S_{n}}{\left(1-\prod_{j=1}^{n}{\left(1-u_{\vartheta (j)}\right)}^{p_{j}}\right)}^{\frac{1}{n!}}\right)\in [0,1],\;{\left(1-\prod_{\vartheta \in S_{n}}{\left(1-\prod_{j=1}^{n}{\left(1-u_{\vartheta (j)}\right)}^{p_{j}}\right)}^{\frac{1}{n!}}\right)}^{\frac{1}{\sum_{j=1}^{n}p_{j}}}\in [0,1],$$
  and
  $$1-{\left(1-\prod_{\vartheta \in S_{n}}{\left(1-\prod_{j=1}^{n}{\left(1-u_{\vartheta (j)}\right)}^{p_{j}}\right)}^{\frac{1}{n!}}\right)}^{\frac{1}{\sum_{j=1}^{n}p_{j}}}\in [0,1].$$
  i.e., 0 ≤ *u* ≤ 1.
  Similarly, we can get 0 ≤ *v* ≤ 1.
  So, condition (i) is met.
(ii) Since *u*_*ϑ*(*j*)_ +*v*_*ϑ*(*j*)_ ≤ 1, then *v*_*ϑ*(*j*)_ ≤ 1 − *u*_*ϑ*(*j*)_, we can get the following inequality
  $$\begin{array}{l} u+v=1-{\left(1-\prod_{\vartheta \in S_{n}}{\left(1-\prod_{j=1}^{n}{\left(1-u_{\vartheta (j)}\right)}^{p_{j}}\right)}^{\frac{1}{n!}}\right)}^{\frac{1}{\sum_{j=1}^{n}p_{j}}}+{\left(1-{\left(\prod_{\vartheta \in S_{n}}\left(1-\prod_{j=1}^{n}v_{\vartheta (j)}^{p_{j}}\right)\right)}^{\frac{1}{n!}}\right)}^{\frac{1}{\sum_{j=1}^{n}p_{j}}}\\ \leq 1-{\left(1-\prod_{\vartheta \in S_{n}}{\left(1-\prod_{j=1}^{n}{\left(1-u_{\vartheta (j)}\right)}^{p_{j}}\right)}^{\frac{1}{n!}}\right)}^{\frac{1}{\sum_{j=1}^{n}p_{j}}}+{\left(1-\prod_{\vartheta \in S_{n}}{\left(1-\prod_{j=1}^{n}{\left(1-u_{\vartheta (j)}\right)}^{p_{j}}\right)}^{\frac{1}{n!}}\right)}^{\frac{1}{\sum_{j=1}^{n}p_{j}}}=1. \end{array}$$
  i.e., 0 ≤ *u* + *v* ≤ 1.
  According to (i) and (ii), we can know the aggregation result from (22) is still an IFN.

**Example 2.** Let (0.5,0.3), (0.7,0,1), and (0.8, 0.2) be three IFNs, and *P* = (1.0,0.5,0.4), then according to (22), we have

$$\begin{array}{l} IFDMM^{\left(1.0,0.5,0.4\right)}\left(\left(0.5,0.3\right),\;\left(0.7,0,1\right),(0.8,0.2)\right)=\\ \left(1-{\left(1-{\left(\prod_{\vartheta \in S_{n}}\left(1-\prod_{j=1}^{n}{\left(1-u_{\vartheta (j)}\right)}^{p_{j}}\right)\right)}^{\frac{1}{n!}}\right)}^{\frac{1}{\sum_{j=1}^{n}p_{j}}},{\left(1-{\left(\prod_{\vartheta \in S_{n}}\left(1-\prod_{j=1}^{n}v_{\vartheta (j)}^{p_{j}}\right)\right)}^{\frac{1}{n!}}\right)}^{\frac{1}{\sum_{j=1}^{n}p_{j}}}\right)\\ (1-(1-(\left(1-{\left(1-0.5\right)}^{1.0}\times {\left(1-0.7\right)}^{0.5}\times {\left(1-0.8\right)}^{0.4}\right)\times \left(1-{\left(1-0.5\right)}^{1.0}\times {\left(1-0.8\right)}^{0.5}\times {\left(1-0.7\right)}^{0.4}\right)\times \\ \left(1-{\left(1-0.7\right)}^{1.0}\times {\left(1-0.5\right)}^{0.5}\times {\left(1-0.8\right)}^{0.4}\right)\times \left(1-{\left(1-0.7\right)}^{1.0}\times {\left(1-0.8\right)}^{0.5}\times {\left(1-0.5\right)}^{0.4}\right)\times \\ {{\left(1-{\left(1-0.8\right)}^{1.0}\times {\left(1-0.5\right)}^{0.5}\times {\left(1-0.7\right)}^{0.4}\right)\times \left(1-{\left(1-0.8\right)}^{1.0}\times {\left(1-0.7\right)}^{0.5}\times {\left(1-0.5\right)}^{0.4}\right))}^{\frac{1}{3!}})}^{\frac{1}{1.0+0.5+0.4}},\\ (1-(\left(1-0.3^{1.0}\times 0.1^{0.5}\times 0.2^{0.4})\times (1-0.3^{1.0}\times 0.2^{0.5}\times 0.1^{0.4})\times (1-0.1^{1.0}\times 0.3^{0.5}\times 0.2^{0.4}\right)\\ {{\times (1-0.1^{1.0}\times 0.2^{0.5}\times 0.3^{0.4})\times (1-0.2^{1.0}\times 0.1^{0.5}\times 0.3^{0.4})\times (1-0.2^{1.0}\times 0.3^{0.5}\times 0.1^{0.4}))}^{\frac{1}{3!}})}^{\frac{1}{1.0+0.5+0.4}})\\ =\left(0.685,0.185\right). \end{array}$$

Similar to the properties of IFMM operator, it is easy to prove some properties of IFDMM operator as follows.

**Property 6 (Idempotency).** If all *α*_*i*_ (*i* = 1,2,…,*n*) are equal, i.e., *α*_*i*_ = *α* = (*u*,*v*), then

$$IFDMM^{P}\left(\alpha_{1},\alpha_{2},\ldots ,\alpha_{n}\right)=\alpha .$$

**Property 7 (Monotonicity).** Let *α*_*i*_ = (*u*_*i*_,*v*_*i*_) and $\alpha_{i}^{\prime }=\left(u_{i}^{\prime },v_{i}^{\prime }\right)$ (*i* = 1,2,…,*n*) be two sets of IFNs. If $u_{i}\geq u_{i}^{\prime },v_{i}\leq v_{i}^{\prime }$ for all *i*, then

$$IFDMM^{P}\left(\alpha_{1},\alpha_{2},\ldots ,\alpha_{n}\right)\geq IFDMM^{P}\left(\alpha_{1}^{\prime },\alpha_{2}^{\prime },\ldots ,\alpha_{n}^{\prime }\right).$$

**Property 8 (Boundedness).** Let *α*_*i*_ = (*u*_*i*_,*v*_*i*_) (*i* = 1,2,…,*n*) be a collections of IFNs, and *α*^−^ = (min(*u*_*i*_),max(*v*_*i*_)), *α*^+^ = (max(*u*_*i*_),min(*v*_*i*_)), Then *α*^−^ ≤ *IFDMM*^*P*^ (*α*_1_,*α*_2_,…,*α*_*n*_) ≤ *α*^+^.

In the following, we will explore some special cases of IFDMM operator with respect to the parameter vector.

1. If *P* = (1,0,⋯,0), the IFDMM reduces to the intuitionistic fuzzy geometric averaging operator [28].
  $$IFDMM^{\left(1,0,\cdots ,0\right)}\left(\alpha_{1},\alpha_{2},\ldots ,\alpha_{n}\right)=\left(\prod_{j=1}^{n}u_{i}^{1/n},1-\prod_{j=1}^{n}{\left(1-v_{i}\right)}^{1/n}\right)$$ (23)
2. If *P* = (*λ*,0,⋯,0), the IFDMM reduces to the intuitionistic fuzzy generalized geometric averaging operator [47].
  $$IFDMM^{\left(\lambda ,0,\cdots ,0\right)}\left(\alpha_{1},\alpha_{2},\ldots ,\alpha_{n}\right)=\left(1-{\left(1-{\prod_{j=1}^{n}\left(1-{\left(1-u_{i}\right)}^{\lambda }\right)}^{1/n}\right)}^{1/\lambda },{\left(1-\prod_{j=1}^{n}{\left(1-v_{i}{}^{\lambda }\right)}^{1/n}\right)}^{1/\lambda }\right)$$ (24)
3. If *P* = (1,1,0,0,⋯,0), the IFDMM reduces to the intuitionistic fuzzy geometric BM operator [48].
  $$IFDMM^{\left(1,1,0,0,\cdots ,0\right)}\left(\alpha_{1},\alpha_{2},\ldots ,\alpha_{n}\right)=\left(1-{\left(1-{\prod_{\begin{array}{l} i,j=1\\ i\neq j \end{array}}^{n}\left(u_{i}+u_{j}-u_{i}u_{j}\right)}^{\frac{1}{n\left(n-1\right)}}\right)}^{1/2},{\left(1-{\prod_{\begin{array}{l} i,j=1\\ i\neq j \end{array}}^{n}\left(1-v_{i}v_{j}\right)}^{\frac{1}{n\left(n-1\right)}}\right)}^{1/2}\right)$$ (25)
4. If $P=\overset{k}{\overset{︷}{(1,1,\cdots ,1}}\overset{n-k}{\overset{︷}{,0,0,\cdots ,0)}}$, the IFDMM reduces to the intuitionistic fuzzy geometric Maclaurin symmetric mean (MSM) operator [43].
  $$IFDMM^{\overset{k}{\overset{︷}{(1,1,\cdots ,1}}\overset{n-k}{\overset{︷}{,0,0,\cdots ,0)}}}\left(\alpha_{1},\alpha_{2},\ldots ,\alpha_{n}\right)=\left(1-{\left(1-{\prod_{1\leq i_{1}≺\ldots ≺i_{k}\leq n}^{}\left(1-\prod_{j=1}^{k}\left(1-u_{i_{j}}\right)\right)}^{1/C_{n}^{k}}\right)}^{1/k},{\left(1-{\prod_{1\leq i_{1}≺\ldots ≺i_{k}\leq n}^{}\left(1-\prod_{j=1}^{k}v_{i_{j}}\right)}^{1/C_{n}^{k}}\right)}^{1/k}\right)$$ (26)
5. If *P* = (1,1,⋯,1), the IFDMM reduces to the intuitionistic fuzzy arithmetic averaging operator [27].
  $$IFDMM^{\left(1,1,\cdots ,1\right)}\left(\alpha_{1},\alpha_{2},\ldots ,\alpha_{n}\right)=\left(1-{\left(\prod_{j=1}^{n}\left(1-u_{j}^{}\right)\right)}^{1/n},\prod_{j=1}^{n}v_{j}^{1/n}\right)$$ (27)
6. If P=(1n,1n,⋯,1n), the IFMM reduces to the intuitionistic fuzzy the arithmetic averaging operator [27].
  IFDMM(1n,1n,⋯,1n)(α1,α2,…,αn)=(1−(∏j=1n(1−uj))1/n,∏j=1nvj1/n) (28)

**Theorem 6.** Let *α*_*i*_ = (*u*_*i*_,*v*_*i*_) (*i* = 1,2,…,*n*) be a collections of IFNs, and *P* = (*p*_1_,*p*_2_,⋯,*p*_*n*_), *Q* = (*q*_1_,*q*_2_,⋯,*q*_*n*_) be two the parameter vectors. If *P* ≺ *Q*, then

$$IFDMM^{P}\left(\alpha_{1},\alpha_{2},\ldots ,\alpha_{n}\right)\leq IFDMM^{Q}\left(\alpha_{1},\alpha_{2},\ldots ,\alpha_{n}\right)$$ (29)

### D. The intuitionistic fuzzy dual weighted MM operator

Similar to IFWMM operator, we will propose intuitionistic fuzzy dual weighted MM (IFDWMM) operator so as to consider the weight vector of the attribute values, which is defined as follows.

**Definition 9.** Let *α*_*i*_ = (*u*_*i*_,*v*_*i*_) (*i* = 1,2,…,*n*) be a collection of IFNs, *w* = (*w*_1_,*w*_2_,…,*w*_*n*_)^*T*^ be the weight vector of *α*_*i*_ (*i* = 1,2,…,*n*), which satisfies *w*_*i*_ ∈ [0,1] and $\sum_{i=1}^{n}w_{i}=1$, and let *P* = (*p*_1_,*p*_2_,⋯,*p*_*n*_) ∈ *R*^*n*^ be a vector of parameters. If

$$IFDWMM^{P}\left(\alpha_{1},\alpha_{2},\ldots ,\alpha_{n}\right)=\frac{1}{\sum_{j=1}^{n}p_{j}}{\left(\prod_{\vartheta \in S_{n}}\sum_{j=1}^{n}\left(p_{j}\alpha_{\vartheta (j)}^{nw_{\vartheta (j)}}\right)\right)}^{\frac{1}{n!}}.$$ (30)

Then we call *IFDWMM*^*P*^ the intuitionistic fuzzy dual weighted MM (IFDMM), where *ϑ*(*j*)(*j* = 1,2,⋯,*n*) is any a permutation of (1,2,⋯,*n*), and *S*_*n*_ is the collection of all permutations of (1,2,⋯,*n*).

**Theorem 7**. Let *α*_*i*_ = (*u*_*i*_,*v*_*i*_) (*i* = 1,2,…,*n*) be a collection of IFNs, then, the result from Definition 9 is also an IFN, and it can be obtained that

$$IFDWMM^{P}\left(\alpha_{1},\alpha_{2},\ldots ,\alpha_{n}\right)=\left(1-{\left(1-\prod_{\vartheta \in S_{n}}{\left(1-\prod_{j=1}^{n}{\left(1-u_{\vartheta (j)}^{nw_{\vartheta (j)}}\right)}^{p_{j}}\right)}^{\frac{1}{n!}}\right)}^{\frac{1}{\sum_{j=1}^{n}p_{j}}},{\left(1-{\left(\prod_{\vartheta \in S_{n}}\left(1-\prod_{j=1}^{n}{\left(1-{\left(1-v_{\vartheta (j)}\right)}^{nw_{\vartheta (j)}}\right)}^{p_{j}}\right)\right)}^{\frac{1}{n!}}\right)}^{\frac{1}{\sum_{j=1}^{n}p_{j}}}\right)$$ (31)

**Proof.** Because $\alpha_{\vartheta (j)}^{nw_{\vartheta (j)}}=\left(u_{\vartheta (j)}^{nw_{\vartheta (j)}},1-{\left(1-v_{\vartheta (j)}\right)}^{nw_{\vartheta (j)}}\right)$, we can replace *u*_*ϑ*(*j*)_ in Eq (22) with $u_{\vartheta (j)}^{nw_{\vartheta (j)}}$, and *v*_*ϑ*(*j*)_ in Eq (22) with $1-{\left(1-v_{\vartheta (j)}\right)}^{nw_{\vartheta (j)}}$, then we can get Eq (31).

Because *α*_*ϑ*(*j*)_ is an IFN, $\alpha_{\vartheta (j)}^{nw_{\vartheta (j)}}$ is also an IFN. By Eq (22), we know *IFDWMM*^*P*^ (*α*_1_,*α*_2_,…,*α*_*n*_) is an IFN.

In the following, we shall explore some desirable properties of IFDWMM operator.

**Property 9 (Monotonicity).** Let *α*_*i*_ = (*u*_*i*_,*v*_*i*_) and $\alpha_{i}^{\prime }=\left(u_{i}^{\prime },v_{i}^{\prime }\right)$ (*i* = 1,2,…,*n*) be two sets of IFNs. If $u_{i}\geq u_{i}^{\prime },v_{i}\leq v_{i}^{\prime }$ for all *i*, then

$$IFDWMM^{P}\left(\alpha_{1},\alpha_{2},\ldots ,\alpha_{n}\right)\geq IFDWMM^{P}\left(\alpha_{1}^{\prime },\alpha_{2}^{\prime },\ldots ,\alpha_{n}^{\prime }\right).$$

**Property 10 (Boundedness).** Let *α*_*i*_ = (*u*_*i*_,*v*_*i*_) (*i* = 1,2,…,*n*) be a collections of IFNs, and *α*^−^ = (min(*u*_*i*_),max(*v*_*i*_)), *α*^+^ = (max(*u*_*i*_),min(*v*_*i*_)), then $\left(u_{\alpha^{-}},v_{\alpha^{-}}\right)\leq IFDWMM^{P}\left(\alpha_{1},\alpha_{2},\ldots ,\alpha_{n}\right)\leq \left(u_{\alpha^{+}},v_{\alpha^{+}}\right)$. Where,

$$u_{\alpha^{-}}=1-{\left(1-{\left(\prod_{\vartheta \in S_{n}}\left(1-\prod_{j=1}^{n}{\left(1-\max{\left(u_{i}\right)}_{}^{nw_{\vartheta (j)}}\right)}^{p_{j}}\right)\right)}^{\frac{1}{n!}}\right)}^{\frac{1}{\sum_{j=1}^{n}p_{j}}},\;v_{\alpha^{-}}={\left(1-{\left(\prod_{\vartheta \in S_{n}}\left(1-\prod_{j=1}^{n}{\left(1-{\left(1-\min(v_{i})\right)}^{nw_{\vartheta (j)}}\right)}^{p_{j}}\right)\right)}^{\frac{1}{n!}}\right)}^{\frac{1}{\sum_{j=1}^{n}p_{j}}},$$

$$u_{\alpha^{+}}=1-{\left(1-{\left(\prod_{\vartheta \in S_{n}}\left(1-\prod_{j=1}^{n}{\left(1-\min{\left(u_{i}\right)}_{}^{nw_{\vartheta (j)}}\right)}^{p_{j}}\right)\right)}^{\frac{1}{n!}}\right)}^{\frac{1}{\sum_{j=1}^{n}p_{j}}},\;v_{\alpha^{+}}={\left(1-{\left(\prod_{\vartheta \in S_{n}}\left(1-\prod_{j=1}^{n}{\left(1-{\left(1-\max(v_{i})\right)}^{nw_{\vartheta (j)}}\right)}^{p_{j}}\right)\right)}^{\frac{1}{n!}}\right)}^{\frac{1}{\sum_{j=1}^{n}p_{j}}}.$$

**Theorem 8.** The IFDMM operator is a special case of the IFDWMM operator.

**Proof.** When $w=\left(\frac{1}{n},\frac{1}{n},\cdots ,\frac{1}{n}\right)$

$$\begin{array}{l} IFDWMM^{P}\left(\alpha_{1},\alpha_{2},\ldots ,\alpha_{n}\right)=\left(1-{\left(1-{\left(\prod_{\vartheta \in S_{n}}\left(1-\prod_{j=1}^{n}{\left(1-u_{\vartheta (j)}^{nw_{\vartheta (j)}}\right)}^{p_{j}}\right)\right)}^{\frac{1}{n!}}\right)}^{\frac{1}{\sum_{j=1}^{n}p_{j}}},{\left(1-{\left(\prod_{\vartheta \in S_{n}}\left(1-\prod_{j=1}^{n}{\left(1-{\left(1-v_{\vartheta (j)}\right)}^{nw_{\vartheta (j)}}\right)}^{p_{j}}\right)\right)}^{\frac{1}{n!}}\right)}^{\frac{1}{\sum_{j=1}^{n}p_{j}}}\right)\\ =\left(1-{\left(1-{\left(\prod_{\vartheta \in S_{n}}\left(1-\prod_{j=1}^{n}{\left(1-u_{\vartheta (j)}^{n\times \frac{1}{n}}\right)}^{p_{j}}\right)\right)}^{\frac{1}{n!}}\right)}^{\frac{1}{\sum_{j=1}^{n}p_{j}}},{\left(1-{\left(\prod_{\vartheta \in S_{n}}\left(1-\prod_{j=1}^{n}{\left(1-{\left(1-v_{\vartheta (j)}\right)}^{n\times \frac{1}{n}}\right)}^{p_{j}}\right)\right)}^{\frac{1}{n!}}\right)}^{\frac{1}{\sum_{j=1}^{n}p_{j}}}\right)\\ =\left(1-{\left(1-{\left(\prod_{\vartheta \in S_{n}}\left(1-\prod_{j=1}^{n}{\left(1-u_{\vartheta (j)}^{}\right)}^{p_{j}}\right)\right)}^{\frac{1}{n!}}\right)}^{\frac{1}{\sum_{j=1}^{n}p_{j}}},{\left(1-{\left(\prod_{\vartheta \in S_{n}}\left(1-\prod_{j=1}^{n}v_{\vartheta (j)}{}^{p_{j}}\right)\right)}^{\frac{1}{n!}}\right)}^{\frac{1}{\sum_{j=1}^{n}p_{j}}}\right)\\ =IFDMM^{P}\left(\alpha_{1},\alpha_{2},\ldots ,\alpha_{n}\right). \end{array}$$

## Two Group Decision Making Approaches Based on the Proposed Operators

In this section, based on the proposed IFWMM or IFDWMM operators, we will develop two novel MAGDM methods, which are described as follows.

Suppose there are *q* decision makers {*X*_1_,*X*_2_,⋯,*X*_*q*_} to evaluate *m* alternatives {*S*_1_,*S*_2_,⋯,*S*_*m*_} with respect to *n* attributes {*A*_1_,*A*_2_,⋯,*A*_*n*_} in a MAGDM problem, where, the weight vector of the attributes is *ω* = (*ω*_1_,*ω*_2_,⋯,*ω*_*n*_) satisfying $\omega_{j}\geq 0(j=1,2,\cdots ,n),\sum_{j=1}^{n}\omega_{j}=1$, and the weight vector of decision makers is *w* = (*w*_1_,*w*_2_,⋯,*w*_*q*_)^*T*^ and satisfying $w_{k}\geq 0,k=1,2,\cdots ,q,\sum_{k=1}^{q}w_{k}=1$. $R_{}^{k}={\left[r_{ij}^{k}\right]}_{m\times n}$ is the given decision matrix of this decision problem, where, $r_{ij}^{k}=\left(u_{ij}^{k},v_{ij}^{k}\right)$ is an IFN given by the decision maker *X*_*k*_ with respect to alternative *S*_*i*_ for attribute *A*_*j*_. Then the goal is to rank the alternatives.

**Fig 1:**
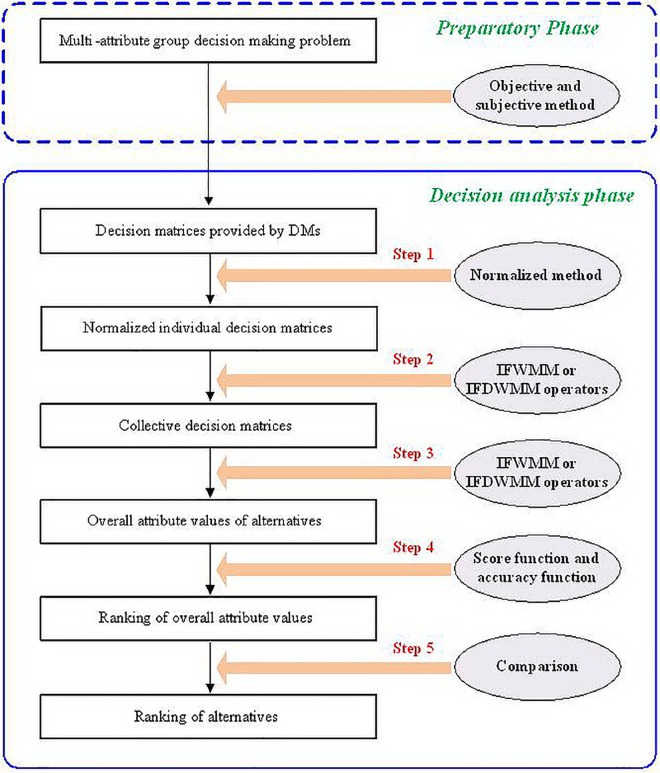
The decision procedure of the proposed MAGDM methods.

In the following, we will use the proposed IFWMM or IFDWMM operators to solve this MAGDM problem and the detailed decision steps are shown as follows (the process of the proposed MAGDM methods is shown in Fig 1.):

**Step 1:** Normalizing the attribute values. In real decision, there exist two types of the attributes which are cost type and benefit types. It is necessary to convert them to the same type so as to give the right decision making. Usually we convert cost type to benefit one by the following formula (Note: The converted attribute value is still expressed by $r_{ij}^{k}$):
  $$r_{ij}^{k}=\left(v_{ij}^{k},u_{ij}^{k}\right)$$ (32)
**Step 2:** Aggregating all individual decision matrix *R*^*k*^ (*k* = 1,2,⋯,*q*) to collective matrix *R* by IFWMM or IFDWMM operators shown as follows:
  $$r_{ij}^{}=IFWMM(r_{ij}^{1},r_{ij}^{2},\cdots ,r_{ij}^{q}),$$ (33)
  or
  $$r_{ij}^{}=IFDWMM(r_{ij}^{1},r_{ij}^{2},\cdots ,r_{ij}^{q}).$$ (34)
**Step 3:** Aggregating all attribute values *r*_*ij*_ (*j* = 1,2,⋯,*n*) to the comprehensive value *Z*_*i*_ by IFWMM or IFDWMM operators shown as follows:
  $$z_{i}=IFWMM(r_{i1}^{},r_{i2}^{},\cdots ,r_{in}^{}),$$ (35)
  or
  $$z_{i}=IFDWMM(r_{i1}^{},r_{i2}^{},\cdots ,r_{in}^{}).$$ (36)
**Step 4:** Ranking *z*_*i*_(*i* = 1,2,⋯,*m*) based on the score function and accuracy function by Definition 4.
**Step 5:** Ranking all the alternatives. The bigger the IFN *z*_*i*_ is, the better the alternative *S*_*i*_ is.

## An Illustrative Example

In order to show the application of the proposed method in this paper, an illustrative example was cited from reference [50] which is about the investment selection decision. An investment corporation wants to select one from five candidate companies {*S*_1_,*S*_2_,*S*_3_,*S*_4_,*S*_5_} to invest some money, in order to make a scientific decision and to avoid loss of investment, three experts {*X*_1_,*X*_2_,*X*_3_} were invited to evaluate the five candidate companies with respect to four attributes {*A*_1_,*A*_2_,*A*_3_,*A*_4_}, where *A*_1_ expresses the risk evaluation, *A*_2_ expresses the growth evaluation, *A*_3_ expresses the social-political impact evaluation and *A*_4_ expresses the environmental impact evaluation. By the existing experience and knowledge, the investment corporation has set the attribute weighting vector *ω* = (0.2,0.1,0.3,0.4)^*T*^ and the experts’ weighting vector *w* = (0.35,0.40,0.25)^*T*^. Three decision makers {*X*_1_,*X*_2_,*X*_3_} give the evaluation matrices shown in Tables 1–3, respectively. In the following, we use the proposed methods in Section 4 to select the best company for the investment corporation.

**Table 1:** Decision matrix *R*^1^ given by decision maker *X*_1_.

	*A*_1_	*A*_2_	*A*_3_	*A*_4_
*S*_1_	(0.5,0.4)	(0.5,0.3)	(0.2,0.6)	(0.4,0.4)
*S*_2_	(0.7,0.3)	(0.7,0.3)	(0.6,0.2)	(0.6,0.2)
*S*_3_	(0.5,0.4)	(0.6,0.4)	(0.6,0.2)	(0.5,0.3)
*S*_4_	(0.8,0.2)	(0.7,0.2)	(0.4,0.2)	(0.5,0.2)
*S*_5_	(0.4,0.3)	(0.4,0.2)	(0.4,0.5)	(0.4,0.6)

**Table 2:** Decision matrix *R*^2^ given by decision maker *X*_2_.

	*A*_1_	*A*_2_	*A*_3_	*A*_4_
*S*_1_	(0.4,0.5)	(0.6,0.2)	(0.5,0.4)	(0.5,0.3)
*S*_2_	(0.5,0.4)	(0.6,0.2)	(0.6,0.3)	(0.7,0.3)
*S*_3_	(0.4,0.5)	(0.3,0.5)	(0.4,0.4)	(0.2,0.6)
*S*_4_	(0.5,0.4)	(0.7,0.2)	(0.4,0.4)	(0.6,0.2)
*S*_5_	(0.6,0.3)	(0.7,0.2)	(0.4,0.2)	(0.7,0.2)

**Table 3:** Decision matrix *R*^3^ given by decision maker *X*_3_.

	*A*_1_	*A*_2_	*A*_3_	*A*_4_
*S*_1_	(0.4,0.2)	(0.5,0.2)	(0.5,0.3)	(0.5,0.2)
*S*_2_	(0.5,0.3)	(0.5,0.3)	(0.6,0.2)	(0.7,0.2)
*S*_3_	(0.4,0.4)	(0.3,0.4)	(0.4,0.3)	(0.3,0.3)
*S*_4_	(0.5,0.3)	(0.5,0.3)	(0.3,0.5)	(0.5,0.2)
*S*_5_	(0.6,0.2)	(0.6,0.4)	(0.4,0.4)	(0.6,0.3)

### A. The decision making steps

To get the best alternative(s), the steps are shown in the following:

**Step 1:** Normalizing the attribute values.
  All the attribute values are the same type, so they do not need to do the standardization.
**Step 2:** Aggregating all individual decision matrix *R*^*k*^ (*k* = 1,2,⋯,*q*) to collective one *R* by IFWMM or IFDWMM operators shown in Tables 4 and 5.

**Table 4:** Collective matrix *R* by IFWMM operator.

	*A*_1_	*A*_2_	*A*_3_	*A*_4_
*S*_1_	(0.422,0.375)	(0.519,0.245)	(0.363,0.452)	(0.456,0.308)
*S*_2_	(0.548,0.342)	(0.579,0.285)	(0.589,0.241)	(0.655,0.241)
*S*_3_	(0.422,0.442)	(0.370,0.442)	(0.448,0.314)	(0.305,0.420)
*S*_4_	(0.572,0.314)	(0.606,0.254)	(0.355,0.396)	(0.519,0.212)
*S*_5_	(0.516,0.273)	(0.540,0.298)	(0.393,0.397)	(0.540,0.405)

**Table 5:** Collective matrix *R* by IFDWMM operator.

	*A*_1_	*A*_2_	*A*_3_	*A*_4_
*S*_1_	(0.445,0.331)	(0.542,0.224)	(0.428,0.405)	(0.479,0.281)
*S*_2_	(0.585,0.323)	(0.613,0.258)	(0.607,0.224)	(0.676,0.224)
*S*_3_	(0.445,0.421)	(0.426,0.421)	(0.484,0.282)	(0.359,0.366)
*S*_4_	(0.637,0.282)	(0.647,0.226)	(0.374,0.339)	(0.542,0.196)
*S*_5_	(0.552,0.256)	(0.591,0.250)	(0.411,0.338)	(0.591,0.324)

**Step 3:** Aggregating all attribute values *r*_*ij*_ (*j* = 1,2,⋯,*n*) to the comprehensive value *Z*_*i*_ by IFWMM or IFDWMM operators shown in Table 6.

**Table 6:** The comprehensive value *Z*_*i*_ by IFWMM and IFDWMM operators.

operator	*S*_1_	*S*_2_	*S*_3_	*S*_4_	*S*_5_
IFWMM	(0.313,0.507)	(0.431,0.456)	(0.270,0.567)	(0.369,0.467)	(0.356,0.502)
IFDWMM	(0.623,0.209)	(0.733,0.176)	(0.586,0.260)	(0.693,0.176)	(0.674,0.199)

**Step 4:** Calculating the score function *S*(*z*_*i*_)(*i* = 1,2,⋯,4) of the collective overall values *z*_*i*_(*i* = 1,2,⋯,5) produced by IFWMM or IFDWMM operators shown in Table 7.

**Table 7:** The score function *S*(*z*_*i*_) of the comprehensive value *Z*_*i*_ by two operators.

operator	*S*_1_	*S*_2_	*S*_3_	*S*_4_	*S*_5_
IFWMM	-0.194	-0.025	-0.297	-0.098	-0.145
IFDWMM	0.413	0.557	0.325	0.517	0.475

**Step 5:** Ranking all the alternatives.

According to the score function *S*(*z*_*i*_)(*i* = 1,2,3,4,5), we can rank the alternatives {*S*_1_,*S*_2_,*S*_3_,*S*_4_,*S*_5_} shown in Table 8.

**Table 8:** The ranking results of five alternatives by two operators.

operator	Ranking results
IFWMM	*S*_2_ ≻ *S*_4_ ≻ *S*_5_ ≻ *S*_1_ ≻ *S*_3_
IFDWMM	*S*_2_ ≻ *S*_4_ ≻ *S*_5_ ≻ *S*_1_ ≻ *S*_3_

From Table 8, we can see that there are the same ranking results by the IFWMM and IFDWMM operators, and the best alternative is *S*_2_.

### B. The influence of the parameter vector P on decision making result of this example

In order to illustrate the influence of the parameter vector *P* on decision making of this example, we set different parameter vector *P* to discuss the ranking results, and the results are shown in Tables 9 and 10.

**Table 9:** Ranking results by utilizing the different parameter vector *P* in the IFWMM operator.

Parameter vector *P*	The score function *S*(*z*_*i*_)	Ranking results
***P*** = (1,0,0,0)	*S*(*z*_1_) = −0.112,*S*(*z*_2_) = 0.138,*S*(*z*_3_) = −0.192,*S*(*z*_4_) = 0.016,*S*(*z*_5_) = −0.064	*S*_2_ ≻ *S*_4_ ≻ *S*_5_ ≻ *S*_1_ ≻ *S*_3_
***P*** = (1,1,0,0)	*S*(*z*_1_) = −0.162,*S*(*z*_2_) = 0.051,*S*(*z*_3_) = −0.238,*S*(*z*_4_) = −0.056,*S*(*z*_5_) = −0.106	*S*_2_ ≻ *S*_4_ ≻ *S*_5_ ≻ *S*_1_ ≻ *S*_3_
***P*** = (1,1,1,0)	*S*(*z*_1_) = −0.180,*S*(*z*_2_) = 0.007,*S*(*z*_3_) = −0.269,*S*(*z*_4_) = −0.081,*S*(*z*_5_) = −0.127	*S*_2_ ≻ *S*_4_ ≻ *S*_5_ ≻ *S*_1_ ≻ *S*_3_
***P*** = (1,1,1,1)	*S*(*z*_1_) = −0.194,*S*(*z*_2_) = −0.025,*S*(*z*_3_) = −0.297,*S*(*z*_4_) = −0.098.*S*(*z*_5_) = −0.145	*S*_2_ ≻ *S*_4_ ≻ *S*_5_ ≻ *S*_1_ ≻ *S*_3_
***P*** = (0.25,0.25,0.25,0.25)	*S*(*z*_1_) = −0.194,*S*(*z*_2_) = −0.025,*S*(*z*_3_) = −0.297,*S*(*z*_4_) = −0.098,*S*(*z*_5_) = −0.145	*S*_2_ ≻ *S*_4_ ≻ *S*_5_ ≻ *S*_1_ ≻ *S*_3_
***P*** = (2,0,0,0)	*S*(*z*_1_) = −0.082,*S*(*z*_2_) = 0.178,*S*(*z*_3_) = −0.157,*S*(*z*_4_) = 0.050,*S*(*z*_5_) = −0.036	*S*_2_ ≻ *S*_4_ ≻ *S*_5_ ≻ *S*_1_ ≻ *S*_3_
***P*** = (3,0,0,0)	*S*(*z*_1_) = −0.049,*S*(*z*_2_) = 0.216,*S*(*z*_3_) = −0.127,*S*(*z*_4_) = 0.084,*S*(*z*_5_) = −0.007	*S*_2_ ≻ *S*_4_ ≻ *S*_5_ ≻ *S*_1_ ≻ *S*_3_

**Table 10:** Ranking results by utilizing the different parameter vector *P* in the IFDWMM operator.

Parameter vector *P*	The score function *S*(*z*_*i*_)	Ranking results
***P*** = (1,0,0,0)	*S*(*z*_1_) = 0.305,*S*(*z*_2_) = 0.514,*S*(*z*_3_) = 0.235,*S*(*z*_4_) = 0.400,*S*(*z*_5_) = 0.373	*S*_2_ ≻ *S*_4_ ≻ *S*_5_ ≻ *S*_1_ ≻ *S*_3_
***P*** = (1,1,0,0)	*S*(*z*_1_) = 0.347,*S*(*z*_2_) = 0.530,*S*(*z*_3_) = 0.283,*S*(*z*_4_) = 0.450,*S*(*z*_5_) = 0.416	*S*_2_ ≻ *S*_4_ ≻ *S*_5_ ≻ *S*_1_ ≻ *S*_3_
***P*** = (1,1,1,0)	*S*(*z*_1_) = 0.378,*S*(*z*_2_) = 0.543,*S*(*z*_3_) = 0.306,*S*(*z*_4_) = 0.485,*S*(*z*_5_) = 0.446	*S*_2_ ≻ *S*_4_ ≻ *S*_5_ ≻ *S*_1_ ≻ *S*_3_
***P*** = (1,1,1,1)	*S*(*z*_1_) = 0.413,*S*(*z*_2_) = 0.557,*S*(*z*_3_) = 0.325,*S*(*z*_4_) = 0.517,*S*(*z*_5_) = 0.475	*S*_2_ ≻ *S*_4_ ≻ *S*_5_ ≻ *S*_1_ ≻ *S*_3_
***P*** = (0.25,0.25,0.25,0.25)	*S*(*z*_1_) = 0.413,*S*(*z*_2_) = 0.557,*S*(*z*_3_) = 0.325,*S*(*z*_4_) = 0.517,*S*(*z*_5_) = 0.475	*S*_2_ ≻ *S*_4_ ≻ *S*_5_ ≻ *S*_1_ ≻ *S*_3_
***P*** = (2,0,0,0)	*S*(*z*_1_) = 0.266,*S*(*z*_2_) = 0.494,*S*(*z*_3_) = 0.200,*S*(*z*_4_) = 0.354,*S*(*z*_5_) = 0.330	*S*_2_ ≻ *S*_4_ ≻ *S*_5_ ≻ *S*_1_ ≻ *S*_3_
***P*** = (3,0,0,0)	*S*(*z*_1_) = 0.234,*S*(*z*_2_) = 0.477,*S*(*z*_3_) = 0.166,*S*(*z*_4_) = 0.316,*S*(*z*_5_) = 0.293	*S*_2_ ≻ *S*_4_ ≻ *S*_5_ ≻ *S*_1_ ≻ *S*_3_

As we can see from Tables 9 and 10, the score functions using the different parameter vector *P* are different, but the ranking results are the same. In general, decision makes can consider some special values, for example, when *P* = (1,0,0,0), the IFWMM operator will become intuitionistic fuzzy weighted average (IFWA) operator [27] and the IFDWMM operator will become intuitionistic fuzzy weighted geometric (IFWG) operator [28]; In addition, for the IFWMM operator, we can find that the more interrelationships of attributes we consider, the smaller value of score functions will become. The parameter vector *P* have greater control ability, the values of score function will become greater. However, for the IFDWMM operator, the result is just the opposite, the more interrelationships of attributes we consider, the greater value of score functions will become. The parameter vector *P* have greater control ability, the values of score function will become small. So, different parameter vector *P* can be regarded as the decision makers’ risk preference.

### C. Comparing with the other methods

To further prove the effectiveness and the prominent advantage of the developed methods in this paper, we solve the same illustrative example by using the three existing MAGDM methods including the intuitionistic fuzzy weighted average (IFWA) operator in [27], the weighted intuitionistic fuzzy Bonferroni mean (WIFBM) operator in [48], and the weighted intuitionistic fuzzy Maclaurin symmetric mean (WIFMSM) operator in [43]. The ranking results are shown in Table 11.

**Table 11:** Ranking results by different methods.

Aggregation operator	Parameter value	Ranking
IFWA [27]	No	*S*_2_ ≻ *S*_4_ ≻ *S*_5_ ≻ *S*_1_ ≻ *S*_3_
WIFBM [48]	*p* = *q* = 1	*S*_2_ ≻ *S*_4_ ≻ *S*_5_ ≻ *S*_1_ ≻ *S*_3_
WIFMSM [43]	*k* = 2	*S*_2_ ≻ *S*_4_ ≻ *S*_5_ ≻ *S*_1_ ≻ *S*_3_
IFWMM in this paper	*P* = (0.25,0.25,0.25,0.25)	*S*_2_ ≻ *S*_4_ ≻ *S*_5_ ≻ *S*_1_ ≻ *S*_3_
IFWDMM in this paper	*P* = (0.25,0.25,0.25,0.25)	*S*_2_ ≻ *S*_4_ ≻ *S*_5_ ≻ *S*_1_ ≻ *S*_3_

From Table 11, we can find that the rank results are same by using five methods. This shows that the new methods in this paper are effective and feasible.

In the following, we will give some comparisons of the three methods and our proposed methods with respect to some characteristic, which are listed in Table 12.

**Table 12:** The comparisons of different methods.

Methods	whether captures interrelationship of two attributes	whether captures interrelationship of multiple attributes	whether makes the method flexible by the parameter vector
IFWA [27]	No	No	No
WIFBM [48]	Yes	No	No
WIFMSM [43]	Yes	Yes	No
IFWMM in this paper	Yes	Yes	Yes
IFWDMM in this paper	Yes	Yes	Yes

According to Table 12 and our further analysis, we can draw the following conclusions.

1. Compared with the method based on the IFWA operator proposed by Xu [27], we can find that the method in [27] can fuse fuzzy information easily, and its calculation process is relatively simple. The weaknesses are (1) the method thinks that the input arguments are independent; (2) this method doesn’t consider the interrelationship among input arguments. However, on the one hand, the new proposed operators in this paper can also consider the interrelationship among all input arguments; on the other hand, they provide a general and flexible aggregation function because they are generalization of most existing aggregation operators. In here, IFWA operator proposed by Xu [27] is a special case of IFWMM operator when the parameter vector *P* = (1,0,…,0,0). Thus, the new method is more general and flexible to solve MAGDM problems than the IFWA operator, especially when the decision makers should consider the interrelationship among the input parameters.
2. Compared with the WIFBM operator in [48], our new methods proposed in this paper can capture interrelationship of multi-input arguments, whereas the WIFBM operator can only capture interrelationship of two arguments. In a realistic decision making environment, maybe there exist interrelationships among two attributes or more than two attributes. Obviously, this is a weakness for the WIFBM operator in [48] because it only considers interrelationship of two arguments. In addition, the IFMM operator can reduce to the intuitionistic fuzzy Bonferroni mean (IFBM) operator when the parameter vector *P* = (1,1,…,0,0).Thus, the new method is more flexible and general to solve MAGDM problems than the above mentioned methods.
3. Compared with the method in [43] based on the WIFMSM operator, although these methods all consider interrelationship of multi-input arguments, the new methods proposed in this paper can make the information aggregation process more flexible by a parameter vector *P*. We have known that the IFMM operator can reduce to the intuitionistic fuzzy Maclaurin symmetric mean (IFMSM) operator [43] when the parameter vector $P=\overset{k}{\overset{︷}{(1,1,\cdots ,1}}\overset{n-k}{\overset{︷}{,0,0,\cdots ,0)}}$. So our method is more universal.

In a word, according to the comparisons and analysis above, the IFWMM operator and the IFDWMM operator proposed in this paper have the advantages (1) they can consider interrelationships among any number of the attributes; (2) they are better and more convenient to deal with the intuitionistic fuzzy information than the existing other methods by a parameter vector *P*.

## Conclusions

Aggregation operators have become a hot issue and an important tool in the decision making fields in recent years. However, they still have some limitations in practical applications. For example, some aggregation operators suppose the attributes are independent of each other. However, the MM operator has a prominent characteristic that it can consider the interaction relationships among any number of attributes by a parameter vector *P*. Inspired by the studies about MM operators, in this paper, we presented some new MM aggregation operators to deal with MAGDM problems under the intuitionistic fuzzy environment, included the intuitionistic fuzzy MM (IFMM) operator, the intuitionistic fuzzy weighted MM (IFWMM) operator, the intuitionistic fuzzy dual MM (IFWMM) operator and the intuitionistic fuzzy dual weighted MM (IFDWMM) operator. Then, the desirable properties were proved and some special cases were discussed. Moreover, we presented two new methods to solve the MAGDM problems with IFNs. Finally, we used an illustrative example to show the feasibility and validity of the new methods by comparing with the other existing methods.

In further research, it is necessary and significant to take the applications of these operators to solve the real decision making problems such as supply chain management, risk assessment, estimate of employment and environmental evaluation, etc. In addition, considering that MM operator has the superiority of compatibility, we can also study some new aggregation operators on the basis of Muirhead mean operator, for example, extend them to linguistic intuitionistic fuzzy sets (LIFSs), Pythagorean fuzzy sets (PFSs), generalized orthopair fuzzy sets [51] and trapezoidal intuitionistic fuzzy numbers [52] and so on.
